# Supplementary material for: Relatively stable pressure effects and time-increasing thermal contraction control Heber geothermal field deformation
Source: Nat Commun. 2024 Jun 17;15:5159. doi: 10.1038/s41467-024-49363-1 (PMC11183258; doi:10.1038/s41467-024-49363-1)
Supplement: Supplementary file 1 — Supplementary Information [file 41467_2024_49363_MOESM1_ESM.pdf]

Supplementary Information for

# Relatively stable pressure effects and time-increasing thermal contraction control Heber geothermal field displacement

Guoyan Jiang[1*], Andrew J. Barbour[2*], Robert J. Skoumal[3], Kathryn Materna[3], Joshua Taron[3] & Aren Crandall-Bear[4]
[1]School of Geodesy and Geomatics, Hubei Luojia Laboratory, Wuhan University, Wuhan, China [2]US Geological Survey, Vancouver, WA, USA. [3]US Geological Survey, Moffett Field, CA, USA. [4]University of Nevada, Reno, NV, USA.
*Corresponding author. E-mail: gyjiang@whu.edu.cn(G.J.); abarbour@usgs.gov(A.J.B.)

**This PDF file includes:**

Supplementary Figs. 1 to 24
Supplementary Tables 1 to 17

# Supplementary figures

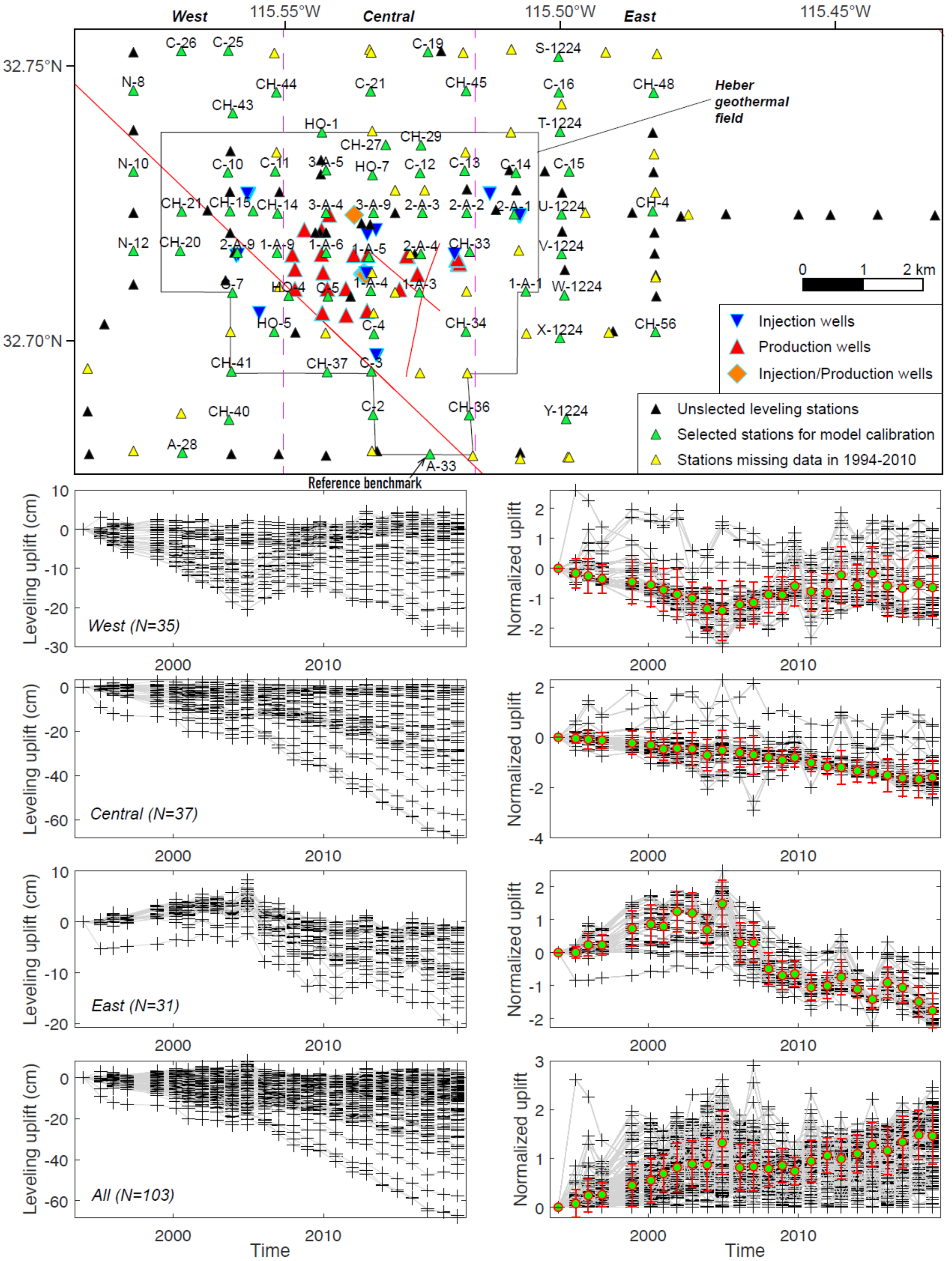


**Supplementary Fig. 1. Spatial distribution of leveling stations and time series of observed vertical displacement.** The study area is divided into three zones: west, central and east. There are only injection wells in the west and east zones. Two panels of the 2nd row show the observed and normalized displacement time series of 35 leveling benchmarks without missing data in the west zone, respectively. The red bars reflect the variability in the normalized leveling time series rather than observation errors. The 3rd

row presents the displacement time series of 37 benchmarks without missing data in the central zone. The 4th row shows the displacement time series of 31 benchmarks without missing data in the east zone. The bottom row shows the time series of 103 benchmarks without missing data in the study area.

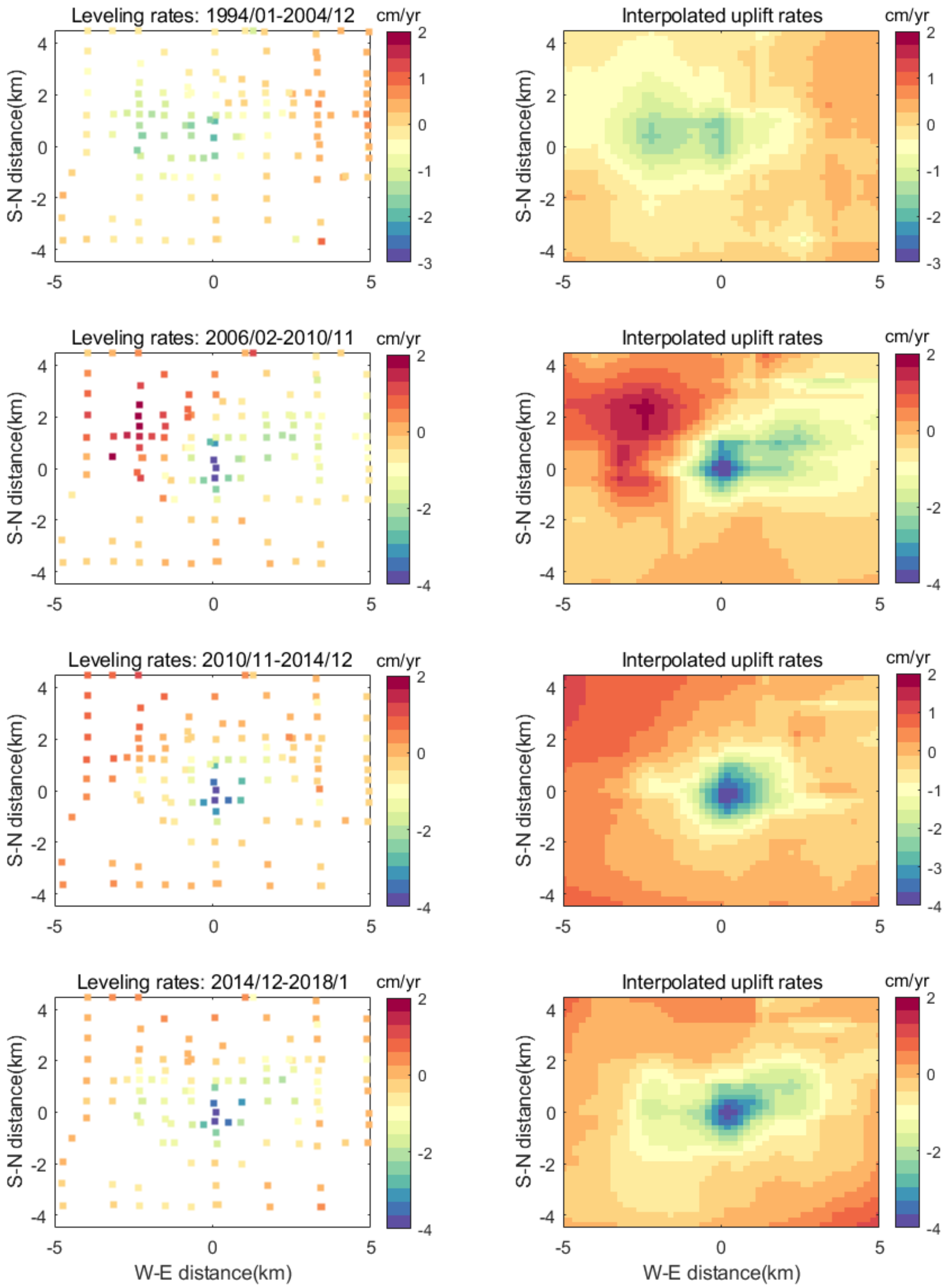


**Supplementary Fig. 2. Vertical displacement rates of four time periods observed by leveling benchmarks.** Four panels of the left column show the displacement rate at each benchmark. The right column shows the corresponding displacement rate maps derived from interpolation with the Kriging method (*Lophaven et al., 2002*).

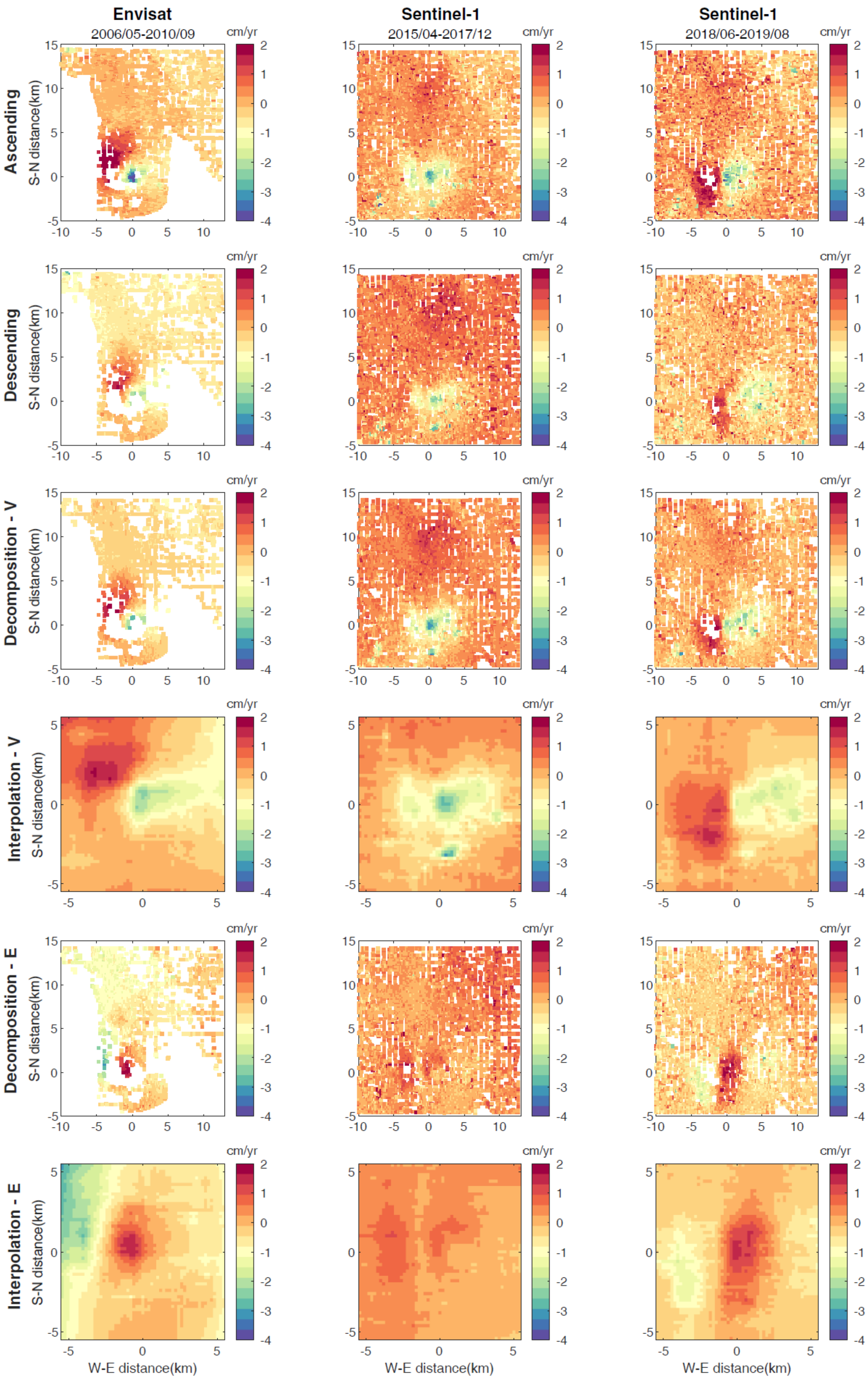
Envisat
2006/05-2010/09
Sentinel-1
2015/04-2017/12
Sentinel-1
2018/06-2019/08
cm/yr
Ascending
Descending
Decomposition - V
Interpolation - V
Decomposition - E
Interpolation - E
S-N distance(km)
W-E distance(km)

**Supplementary Fig. 3. InSAR measured surface displacement rates.** The displacement within three time periods is first measured with the ascending and descending track SAR images of the Envisat and Sentinel-1 satellites in the line-of-sight (LOS) direction and then decomposed into the vertical and eastern components. Note the change in spatial scales between the observed and interpolated results.

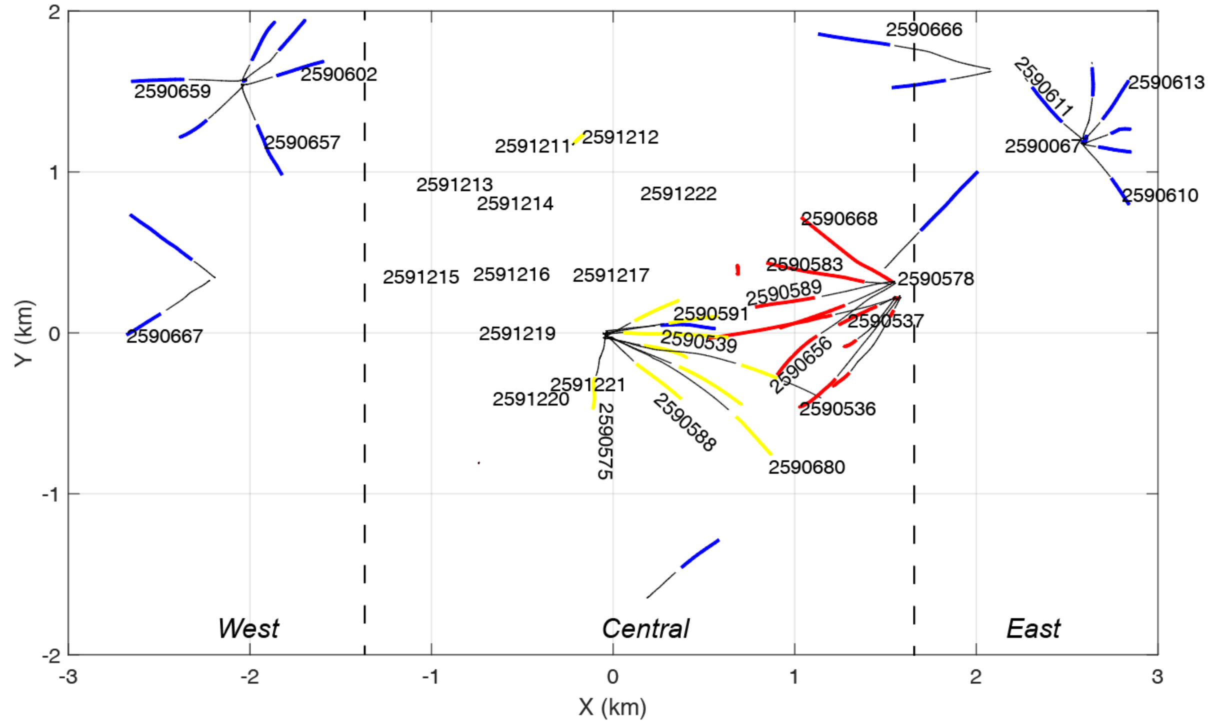


**Supplementary Fig. 4. Top view of the trajectories of operation wells within the HGF.** Blue color marks the open-hole sections of injection wells. Red color labels the open-hole sections of extraction wells. Yellow color shows the open-hole sections of 10 wells that were first completed for extraction and later converted to injection. The wells selected for model calibration are labeled with their California API IDs.

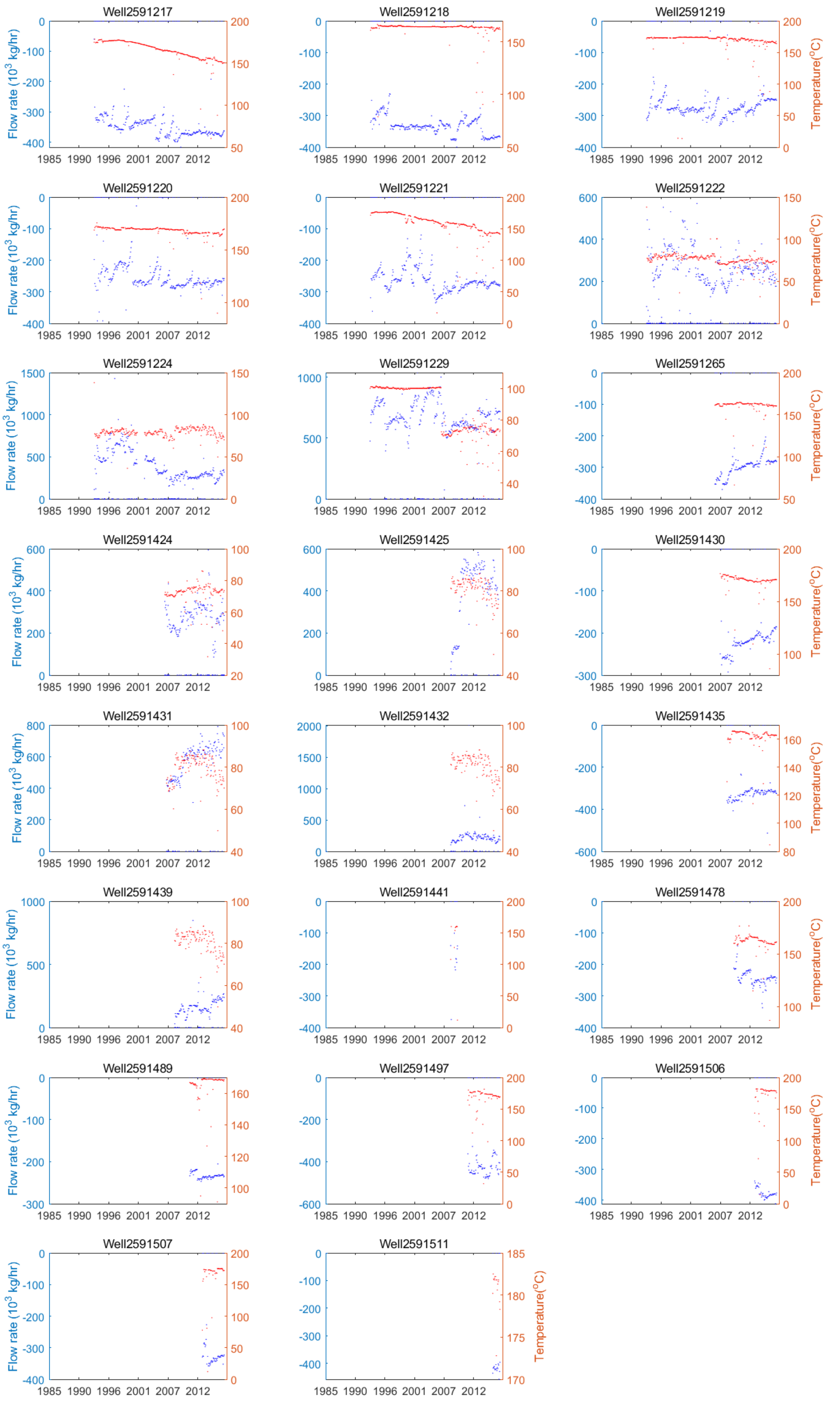


**Supplementary Fig. 5. Production rates (blue) and temperature records (red) of 69 wells.**

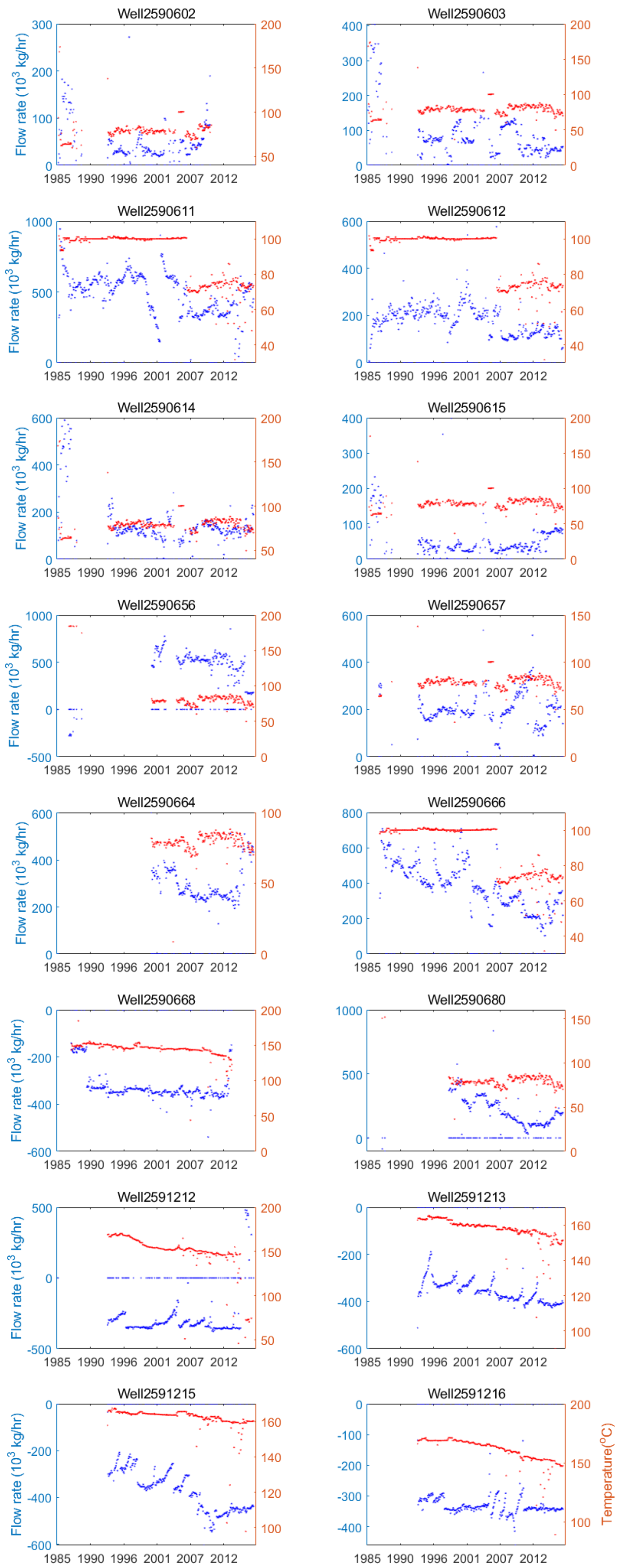


**Supplementary Fig. 5. Continued.**

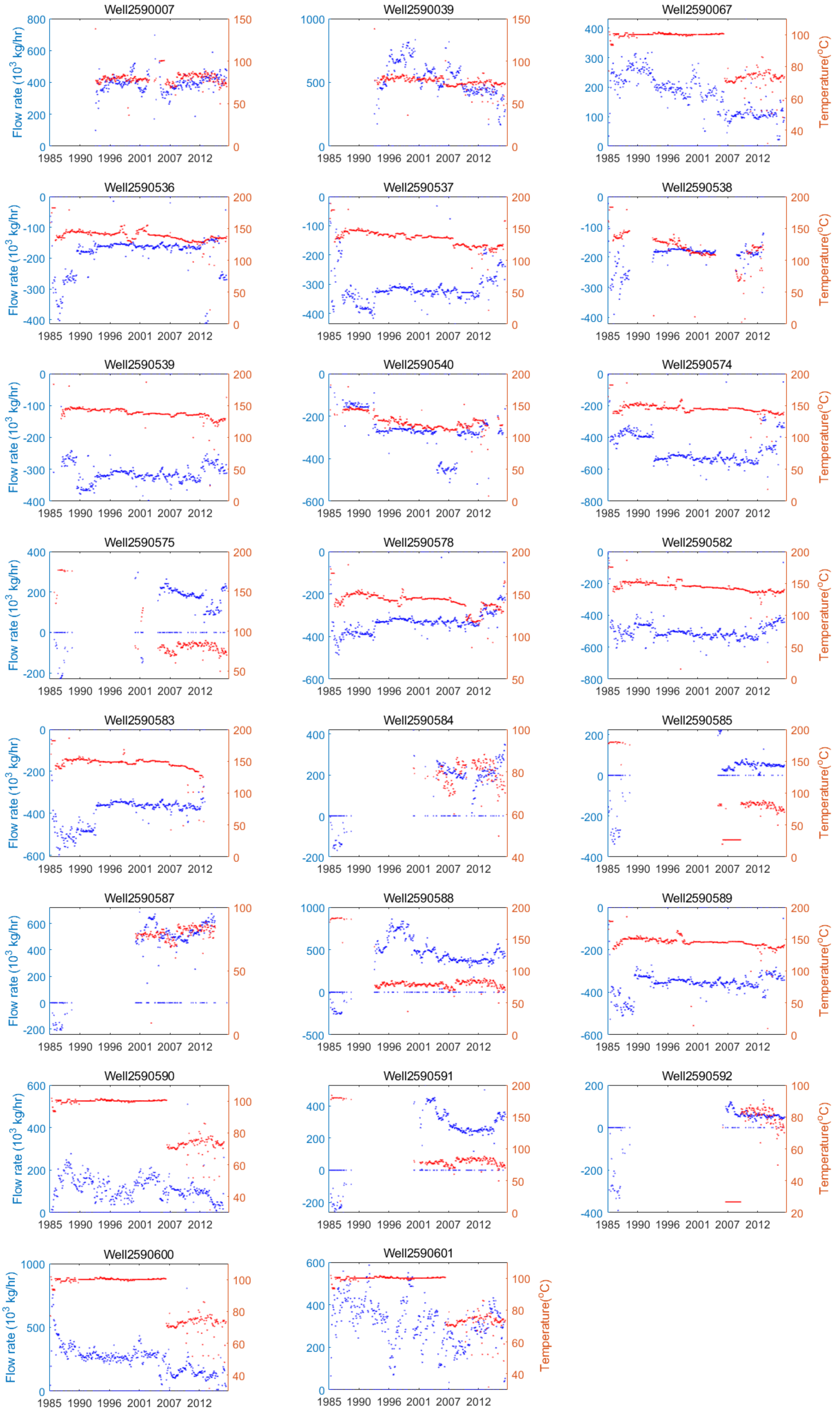


**Supplementary Fig. 5. Continued.**

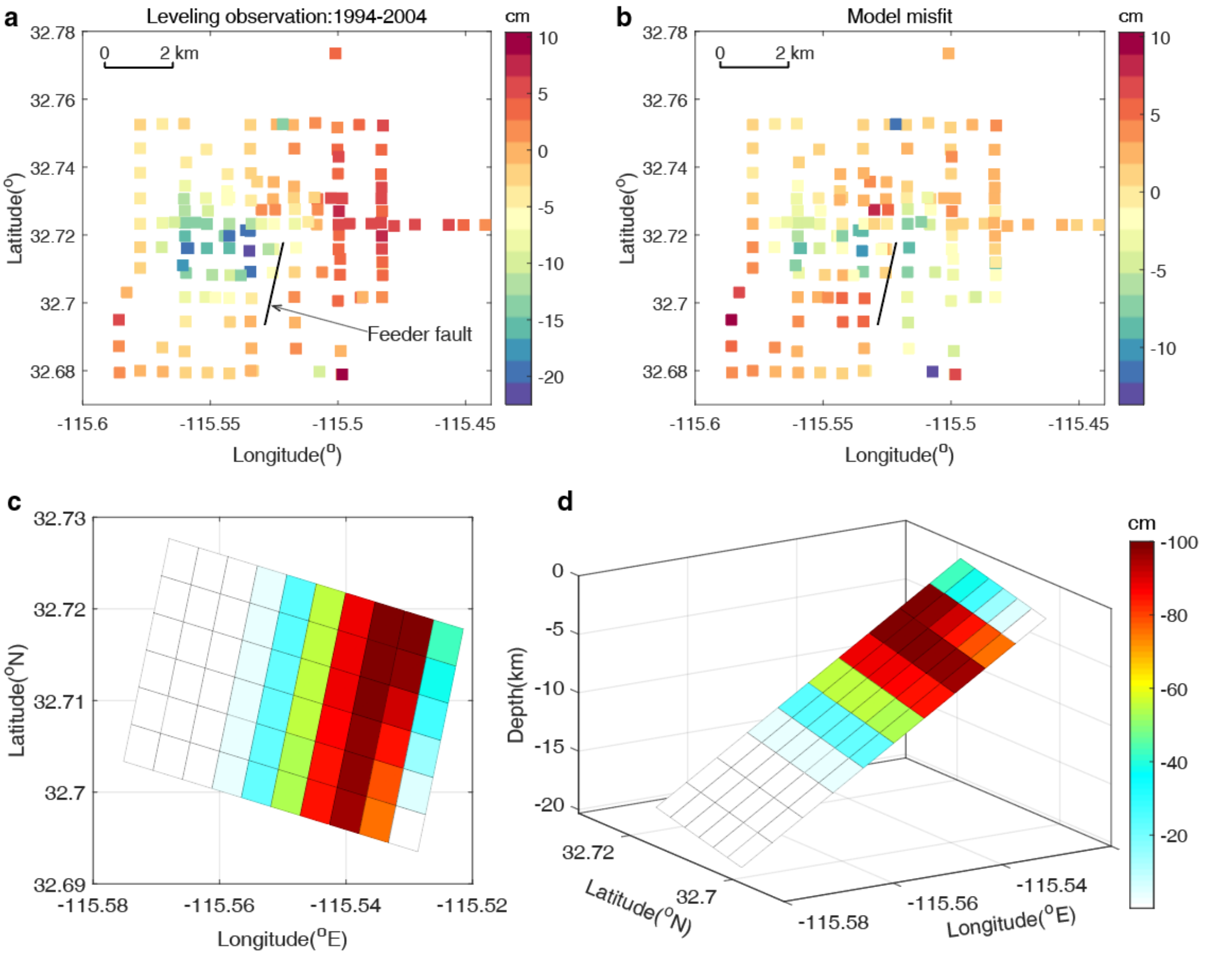


**Supplementary Fig. 6. Fault modeling of long-term vertical surface displacement from 1994 to 2004.** **a** Leveling observed surface displacement from January 1994 to December 2004. **b** Misfit between leveling observations and predictions with the inverted slip distribution on the normal-slip feeder fault shown in **c** (2D view) and **d** (3D view). Negative values represent normal slip.

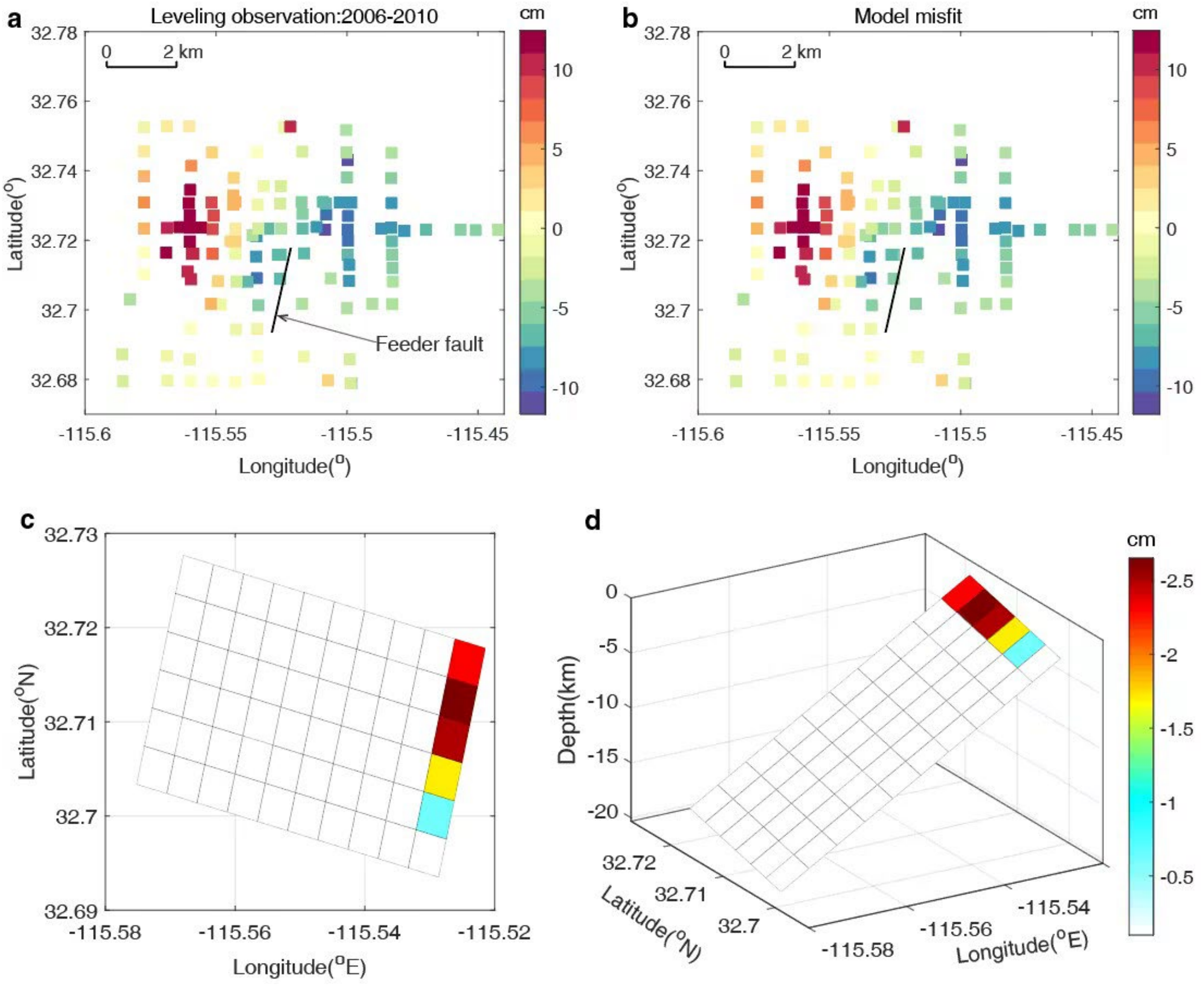


**Supplementary Fig. 7. Fault modeling of vertical transient displacement from 2006 to 2010. a** Corrected leveling displacement with removing the long-term displacement trends observed before 2005. **b** Misfit between leveling observations and predictions with the inverted slip distribution on the normal-slip feeder fault shown in **c** (2D view) and **d** (3D view). Negative values represent normal slip.

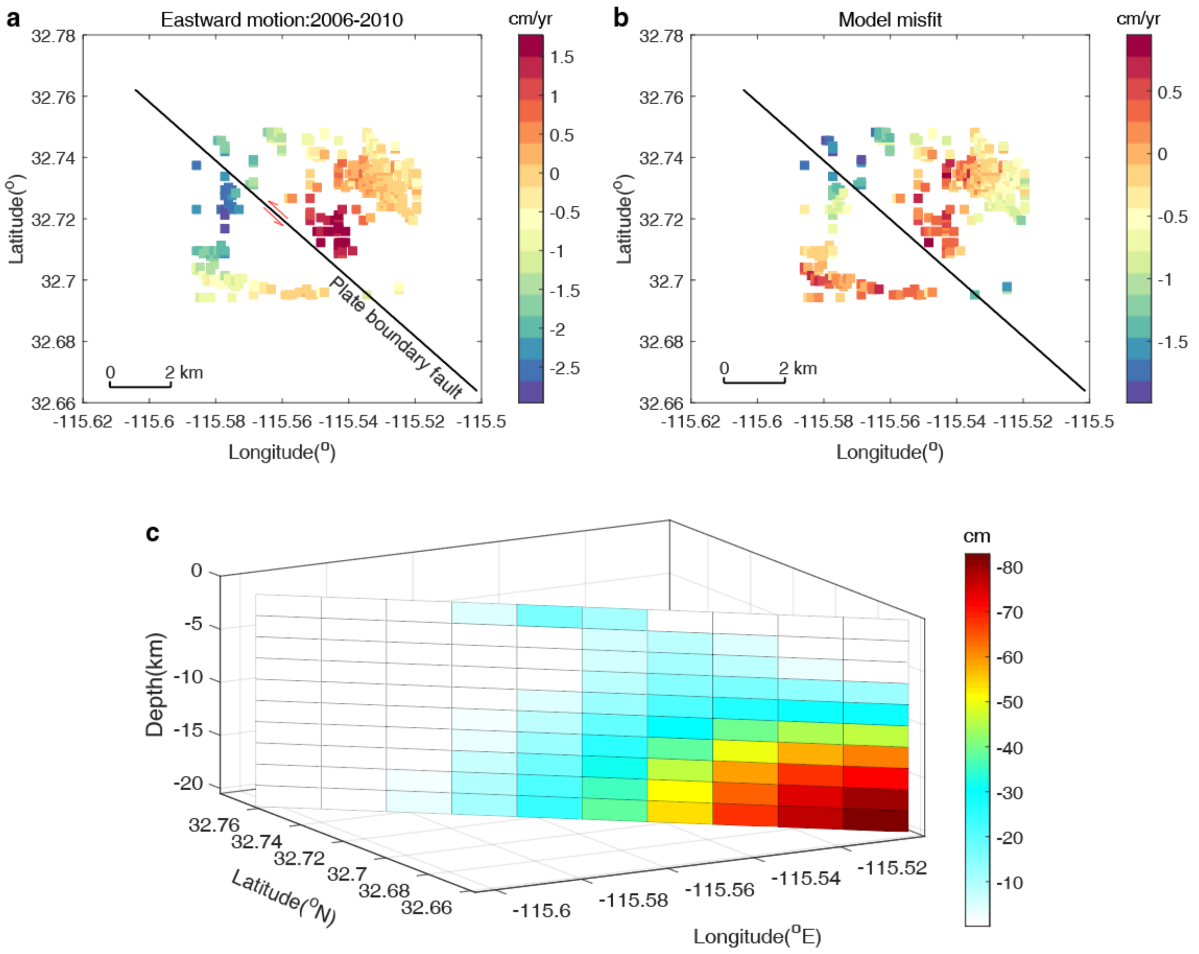


**Supplementary Fig. 8. Fault modeling of the eastward surface displacement from 2006 to 2010 derived from InSAR observations. a** Observed surface displacement in the east direction. **b** Misfit between leveling observations and predictions with the inverted slip distribution on the strike-slip plate boundary fault shown in **c**. Negative values represent right-lateral slip.

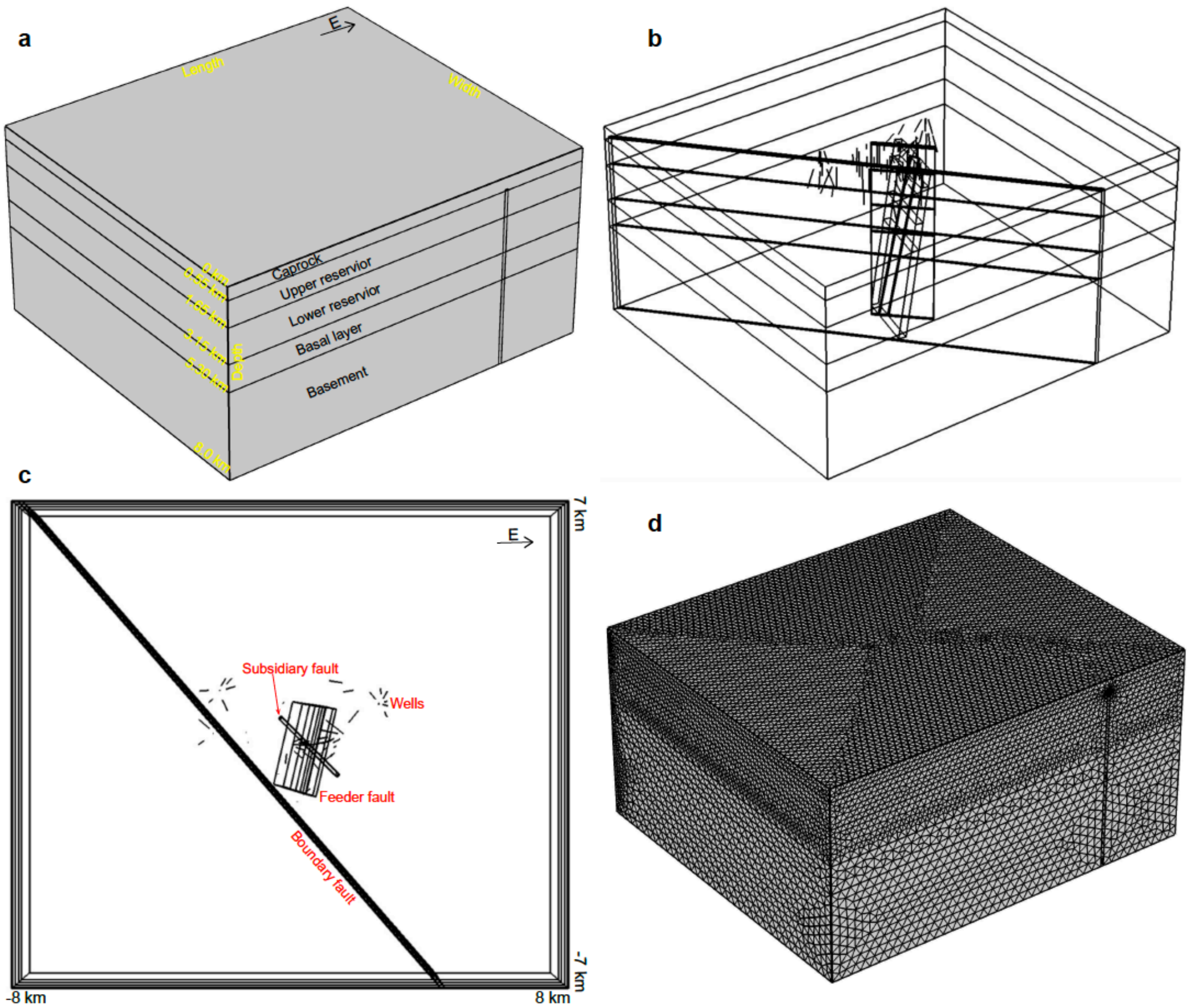


**Supplementary Fig. 9. 3D geometrical model of the HGF. a** Stratigraphic division**; b** Perspective view; **c** Top view with faults and wells labeled; **d** Tetrahedral model mesh.

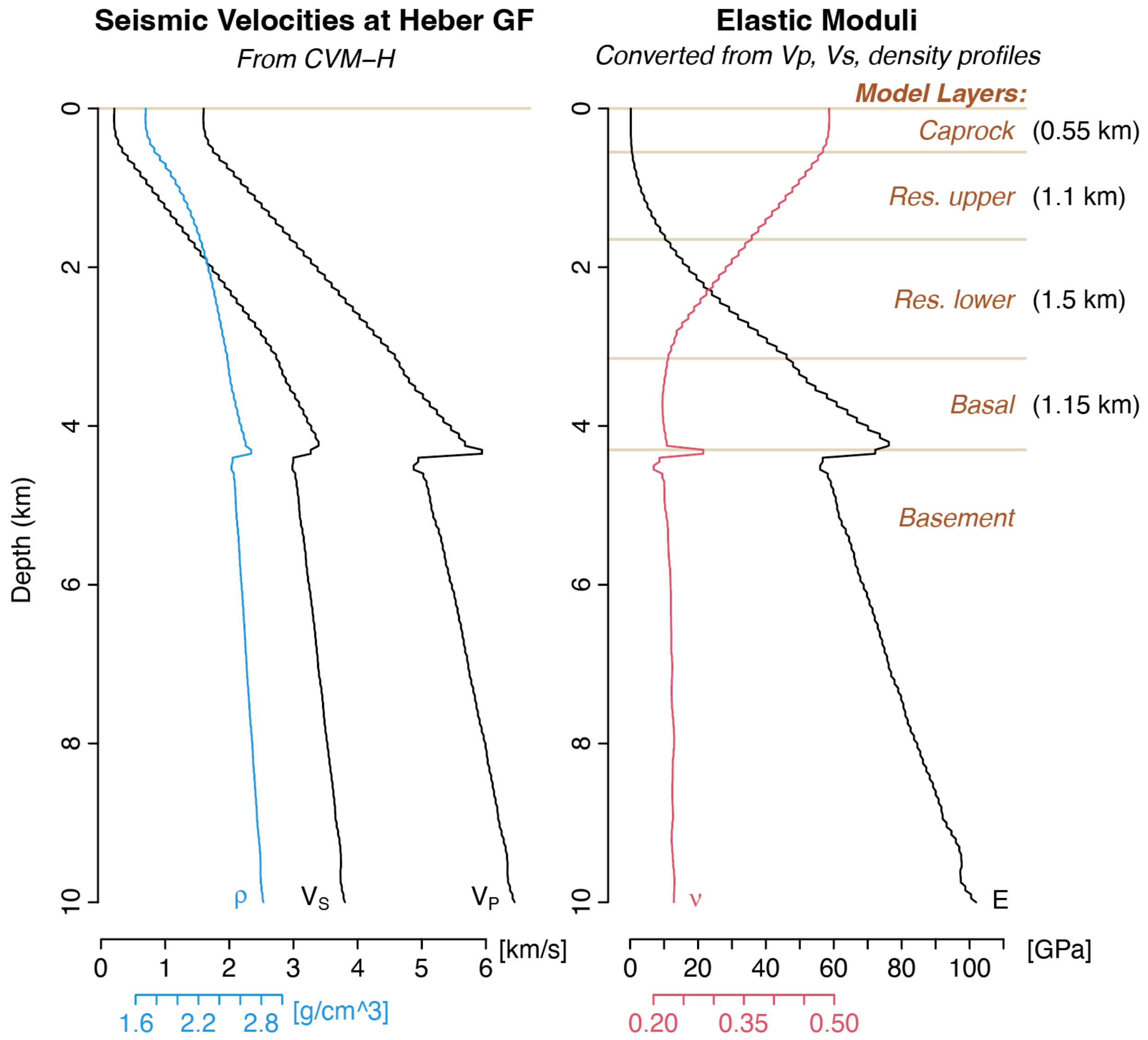


**Supplementary Fig. 10. Profiles of seismic velocities ($V_p$ and $V_s$) and density ($\rho$) at the HGF (left) converted to Poisson ratio ($\nu$) and Young's modulus ($E$) (right).** The velocities are from the SCEC Community Velocity Model-Harvard (CVM-H) (*Shaw et al., 2015*). The depths of the five model layer interfaces are shown as horizontal lines.

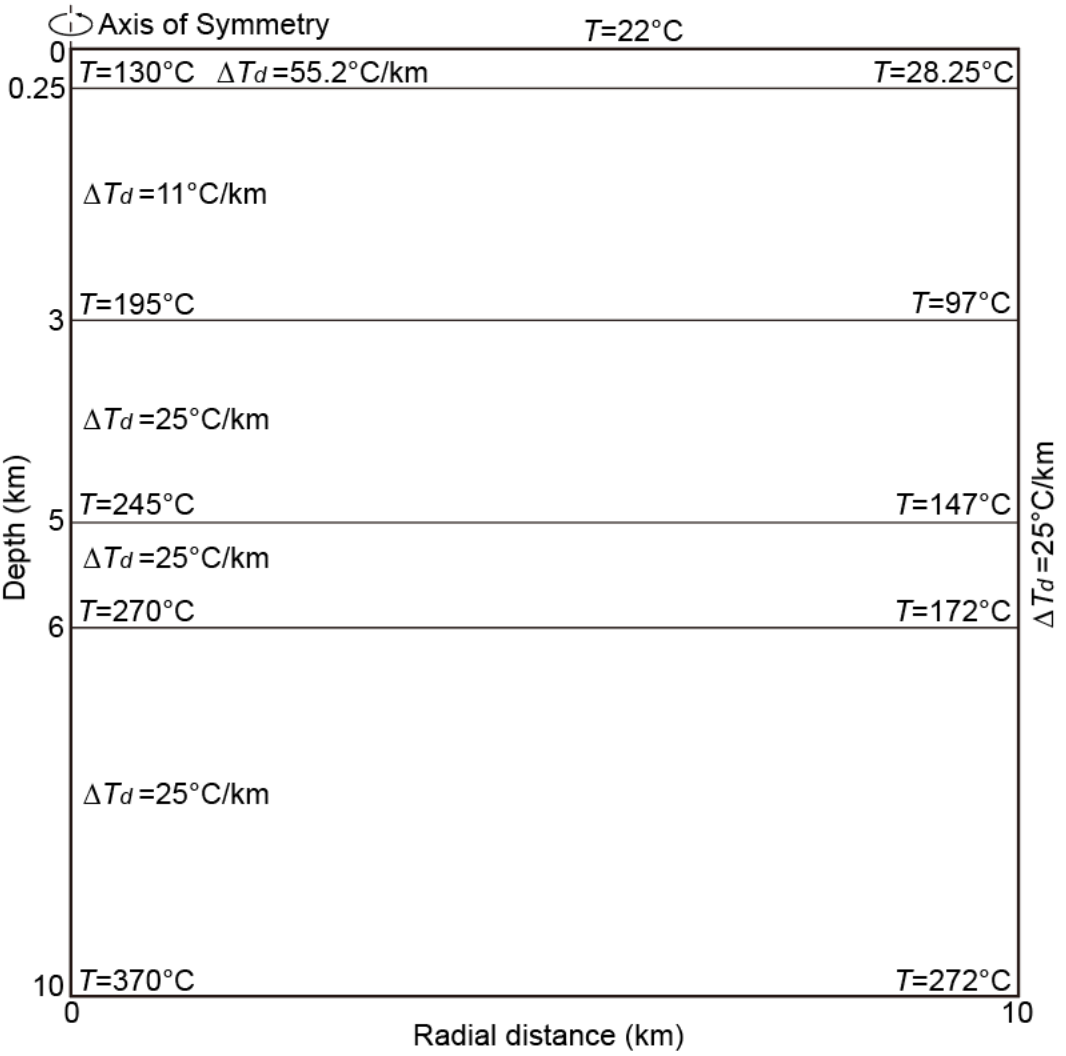


**Supplementary Fig. 11. Initial temperature distribution model of the HGF for 3D thermo-hydro-mechanical (THM) simulation.**

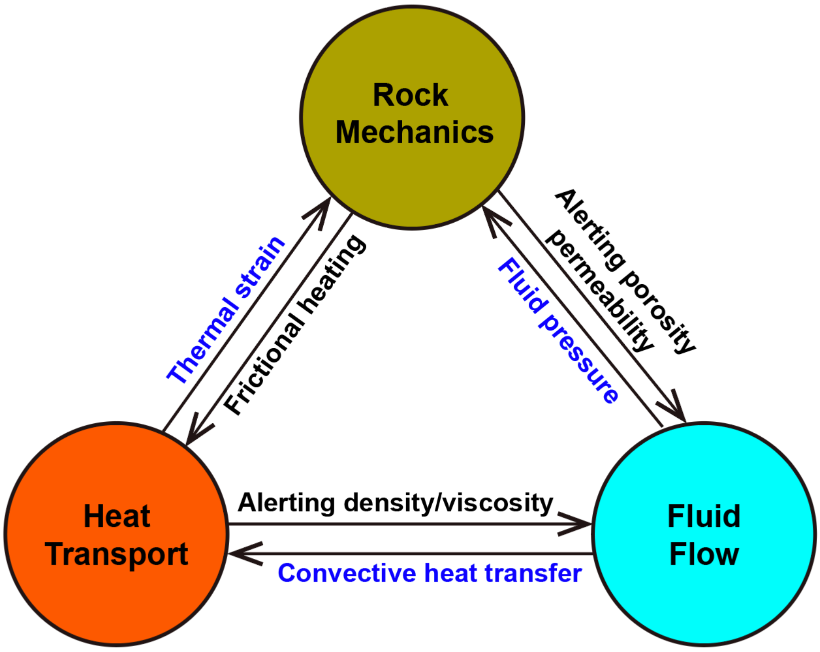


**Supplementary Fig. 12. Flowchart of thermo-hydro-mechanical coupling.** The arrows labeled with blue words show the effects included in our simulations.

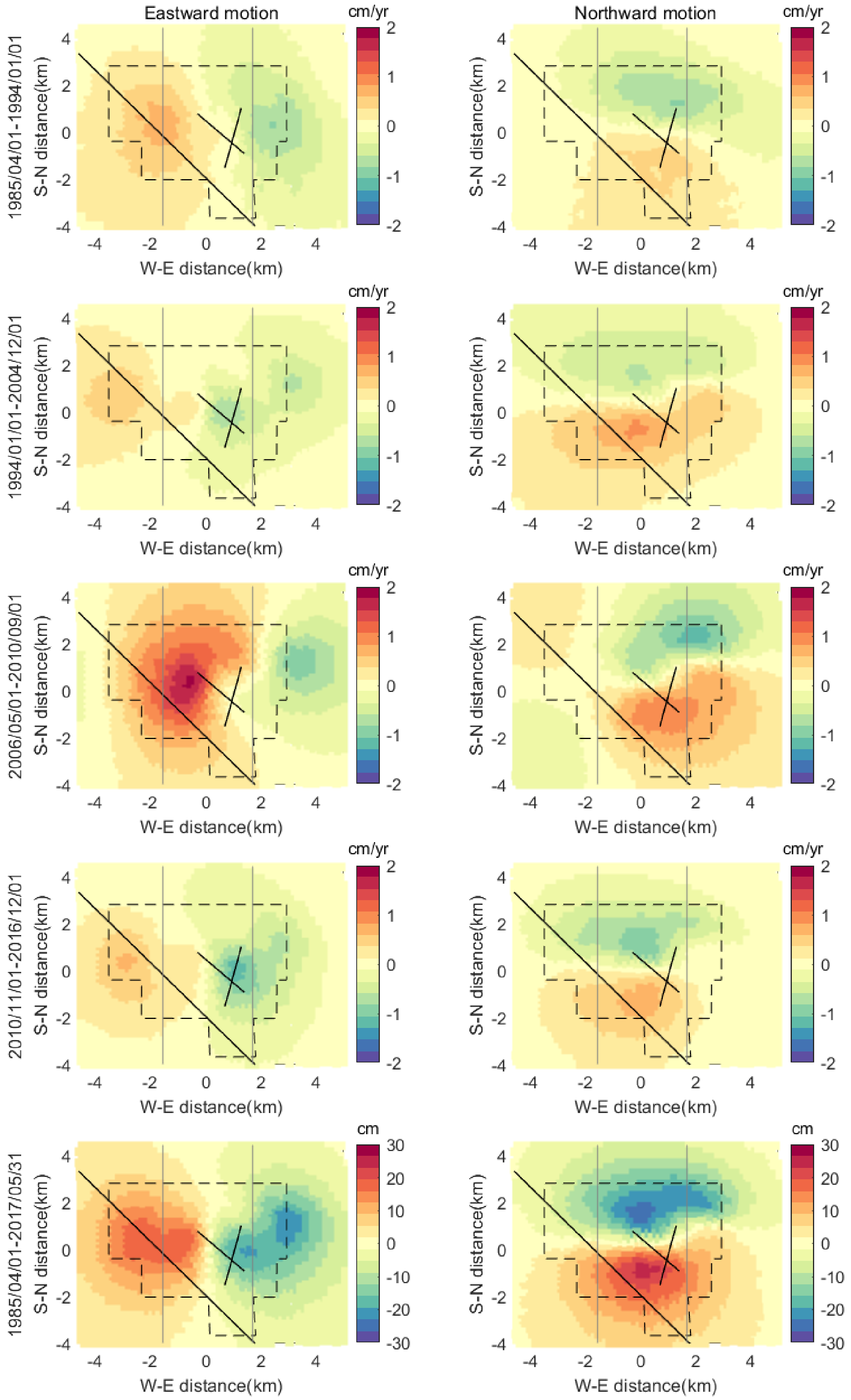


**Supplementary Fig. 13. Simulated horizontal surface displacement with the calibrated model in five time periods.** Eastward and northward motions are defined to be positive.

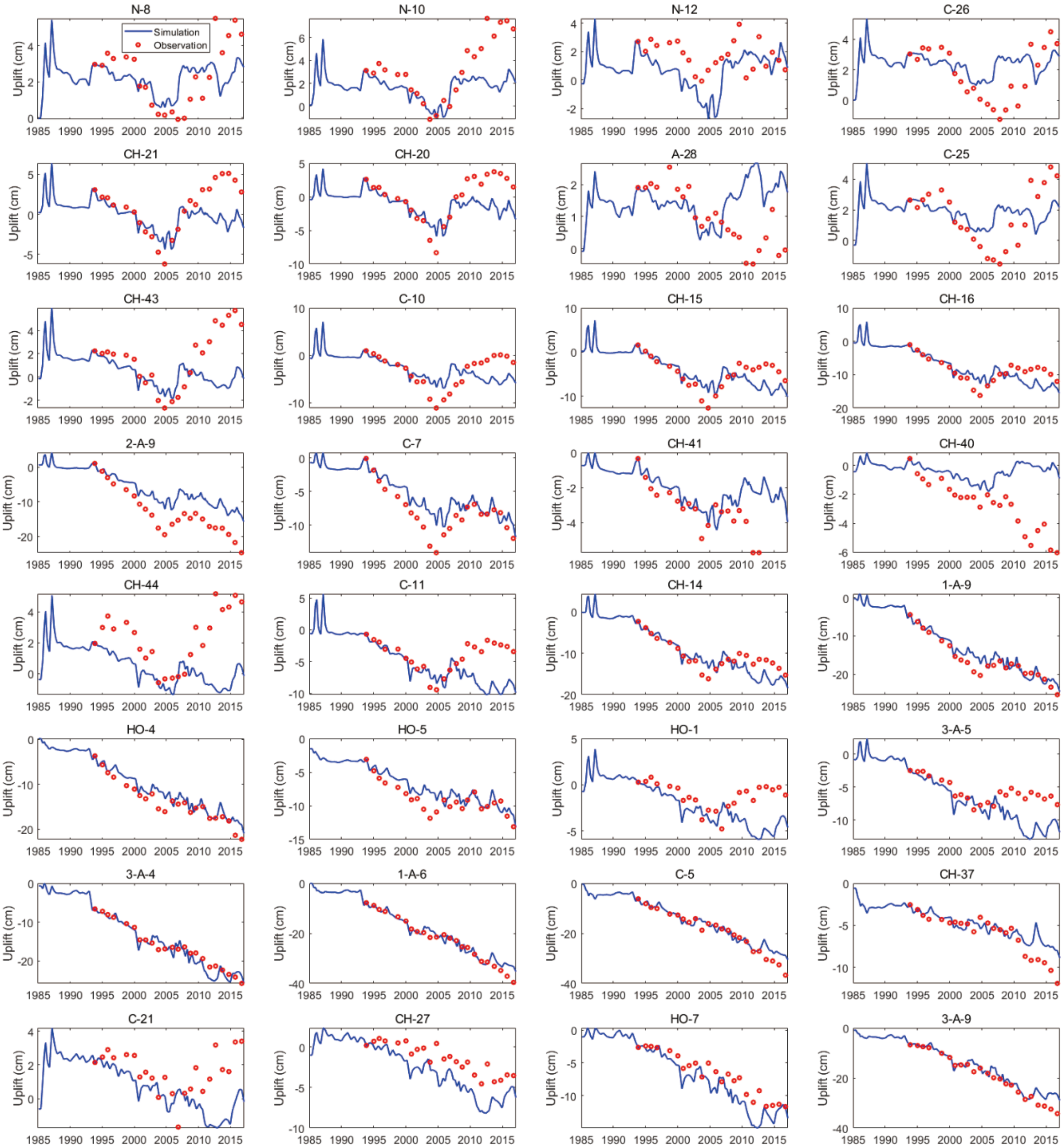


**Supplementary Fig. 14. Comparison of the vertical displacement time series observed at 64 selected benchmarks with the time series simulated by the calibrated model.**

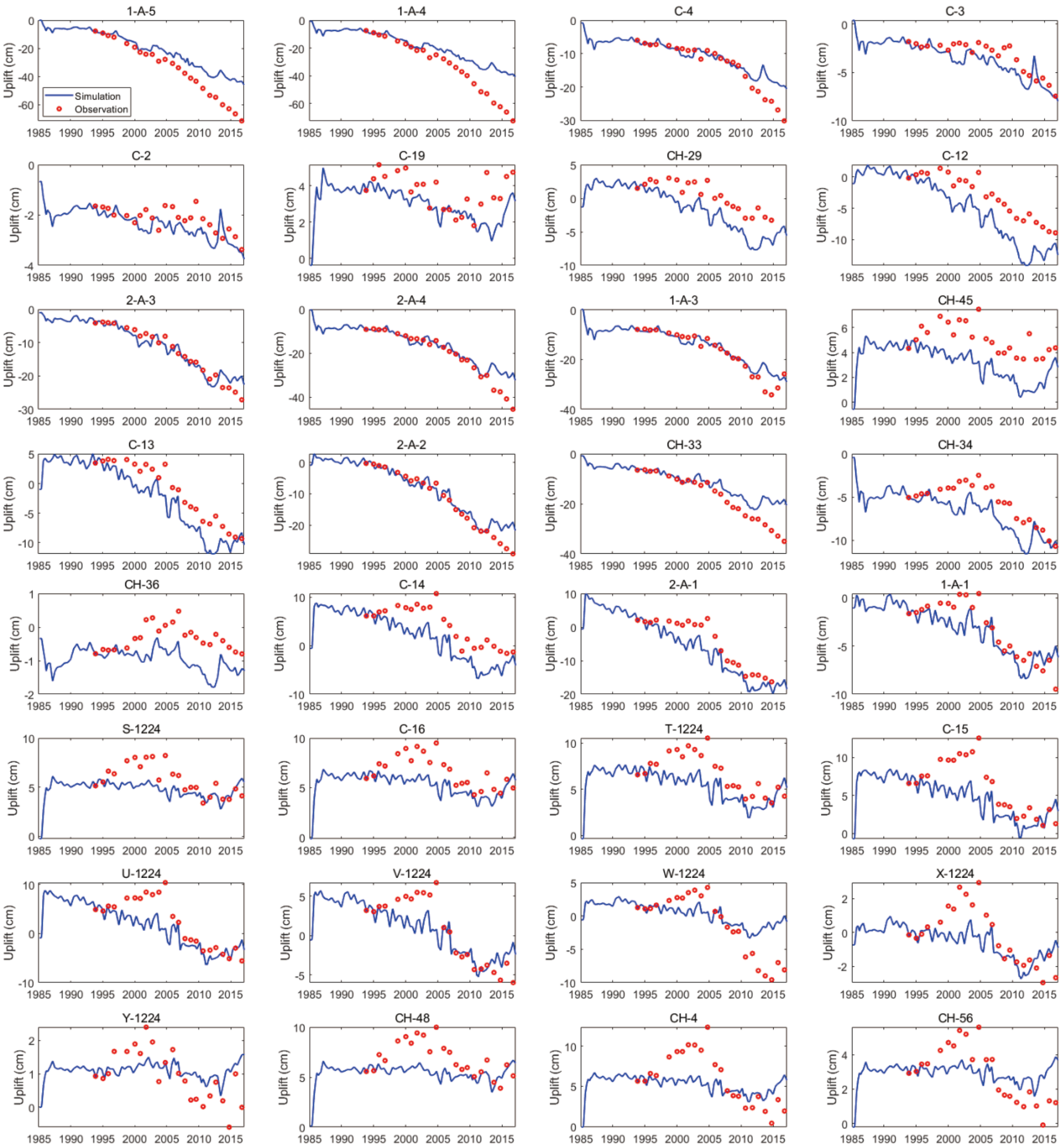


**Supplementary Fig. 14. Continued.**

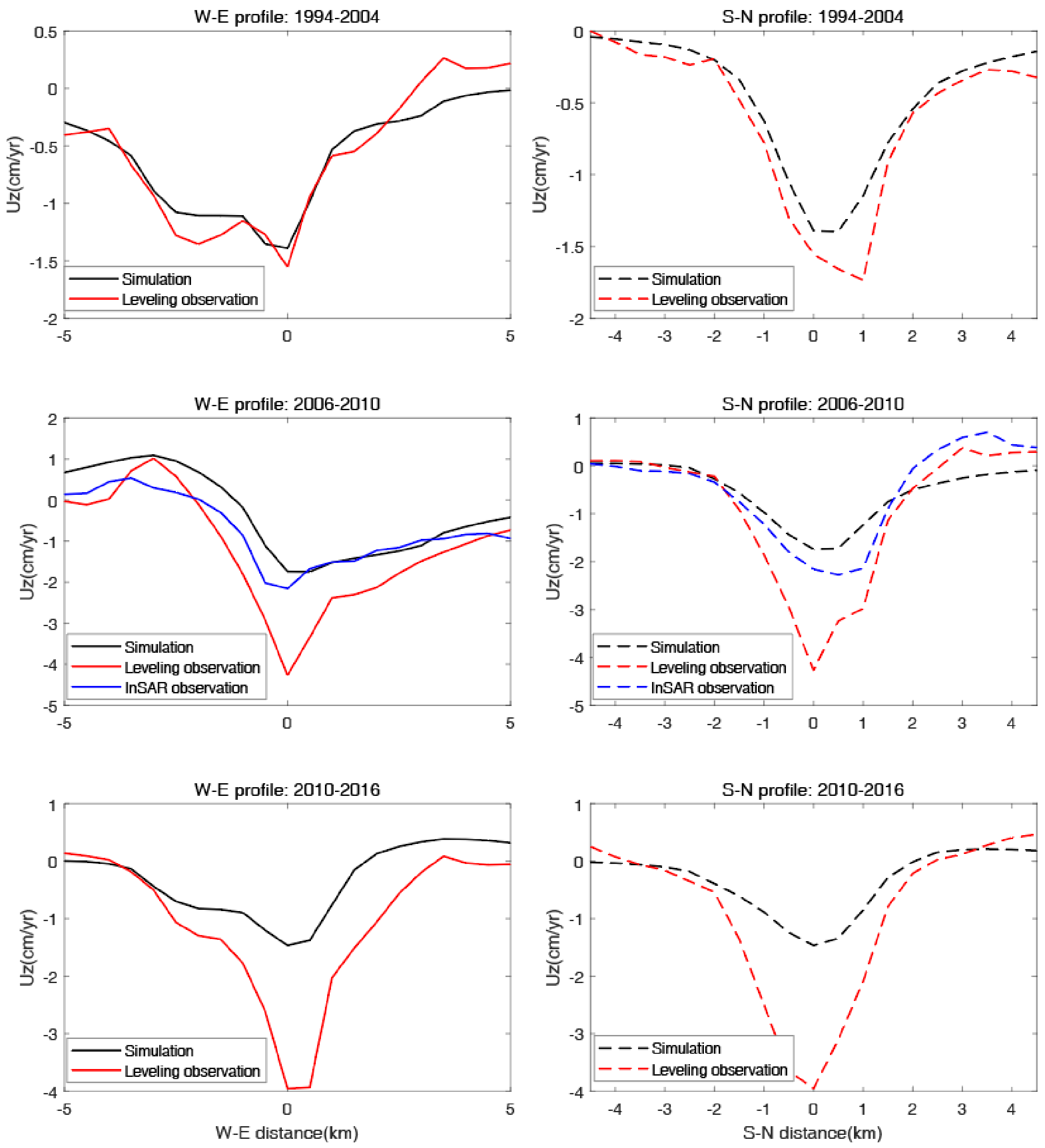


**Supplementary Fig. 15. Comparison of simulated vertical displacement with the observations along the W-E and S-N profiles.** Each profile goes through the center of the HGF.

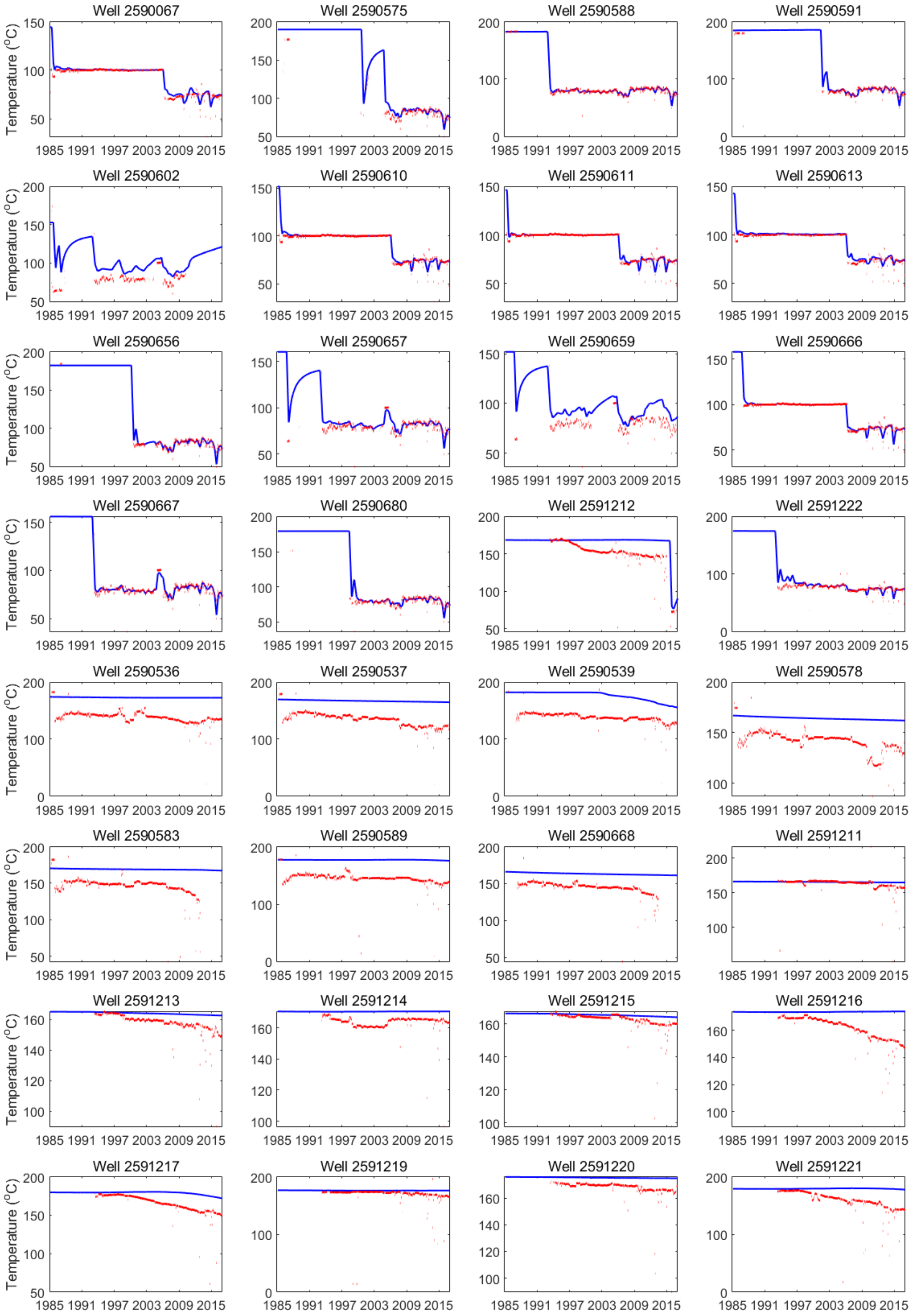


**Supplementary Fig. 16. Comparison of simulated temperature variations with the records of 32 selected wells.** Red points: reported fluid temperature; blue curves: simulated well temperature with the calibrated model.

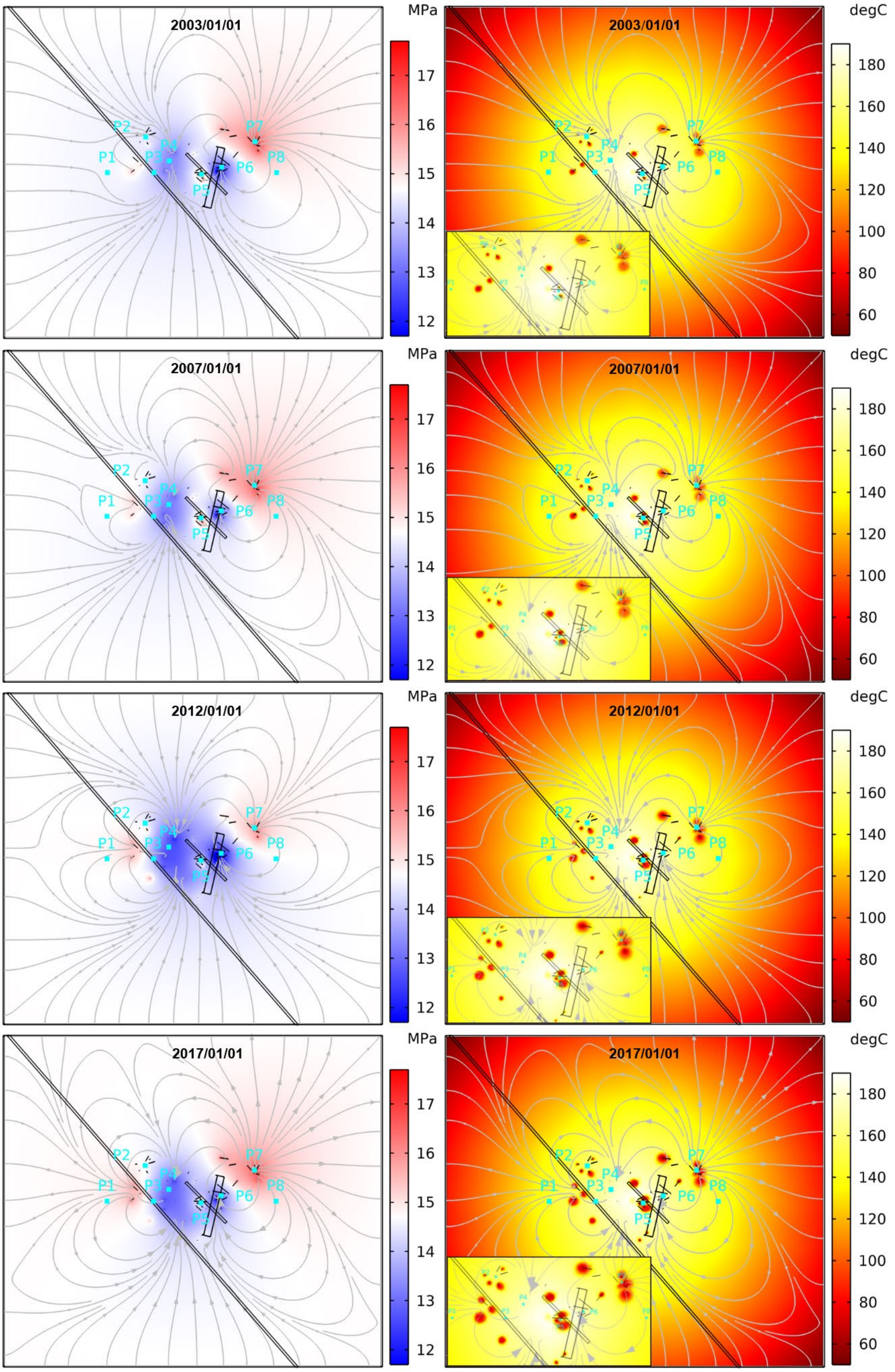


**Supplementary Fig. 17. Simulated pressure and temperature distribution with the calibrated model at the bottom of the upper reservoir layer (depth=1.5 km).** Gray curves and arrows showing the movement of fluid.

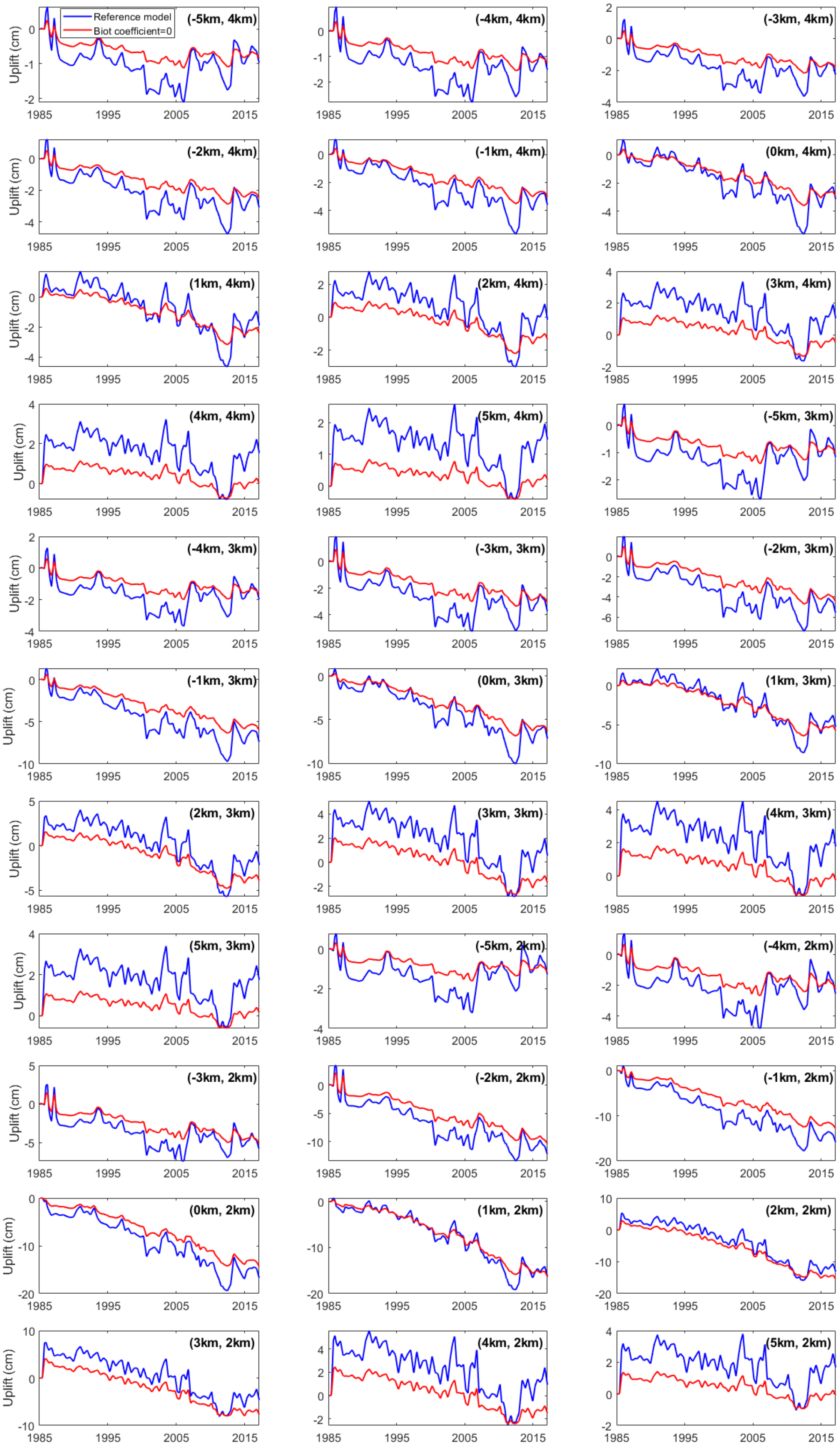
Reference model
Biot coefficient=0
Uplift (cm)
1985
1995
2005
2015
(-5km, 4km)
(-4km, 4km)
(-3km, 4km)
(-2km, 4km)
(-1km, 4km)
(0km, 4km)
(1km, 4km)
(2km, 4km)
(3km, 4km)
(4km, 4km)
(5km, 4km)
(-5km, 3km)
(-4km, 3km)
(-3km, 3km)
(-2km, 3km)
(-1km, 3km)
(0km, 3km)
(1km, 3km)
(2km, 3km)
(3km, 3km)
(4km, 3km)
(5km, 3km)
(-5km, 2km)
(-4km, 2km)
(-3km, 2km)
(-2km, 2km)
(-1km, 2km)
(0km, 2km)
(1km, 2km)
(2km, 2km)
(3km, 2km)
(4km, 2km)
(5km, 2km)

**Supplementary Fig. 18. Vertical surface displacement at 99 points simulated with the calibrated (reference) model and the model with the Biot coefficients of the two reservoirs and the feeder fault equal to 0.** Locations of the points relative to the center of the model are labeled within each panel.

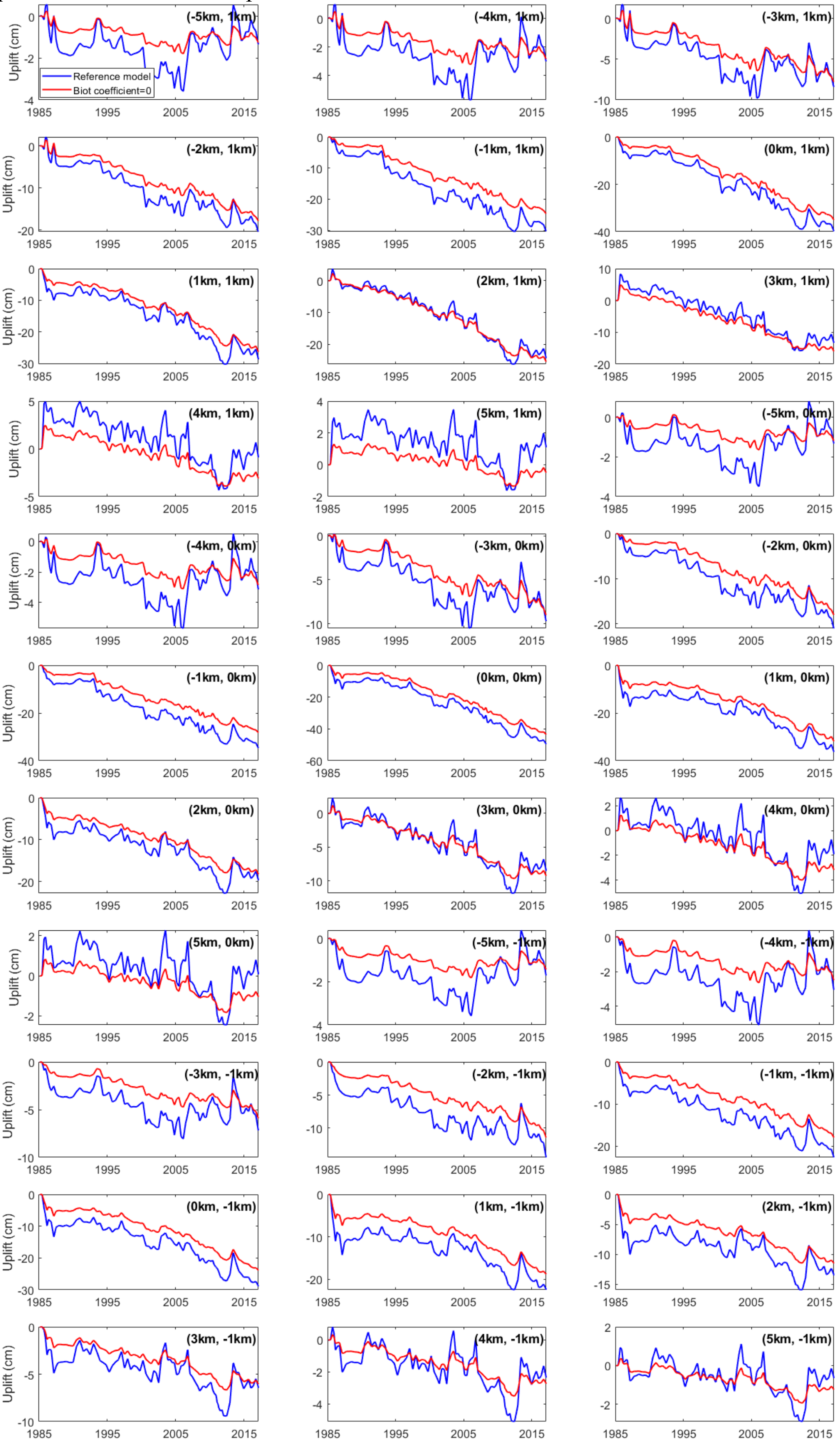

**Supplementary Fig. 18. Continued.**

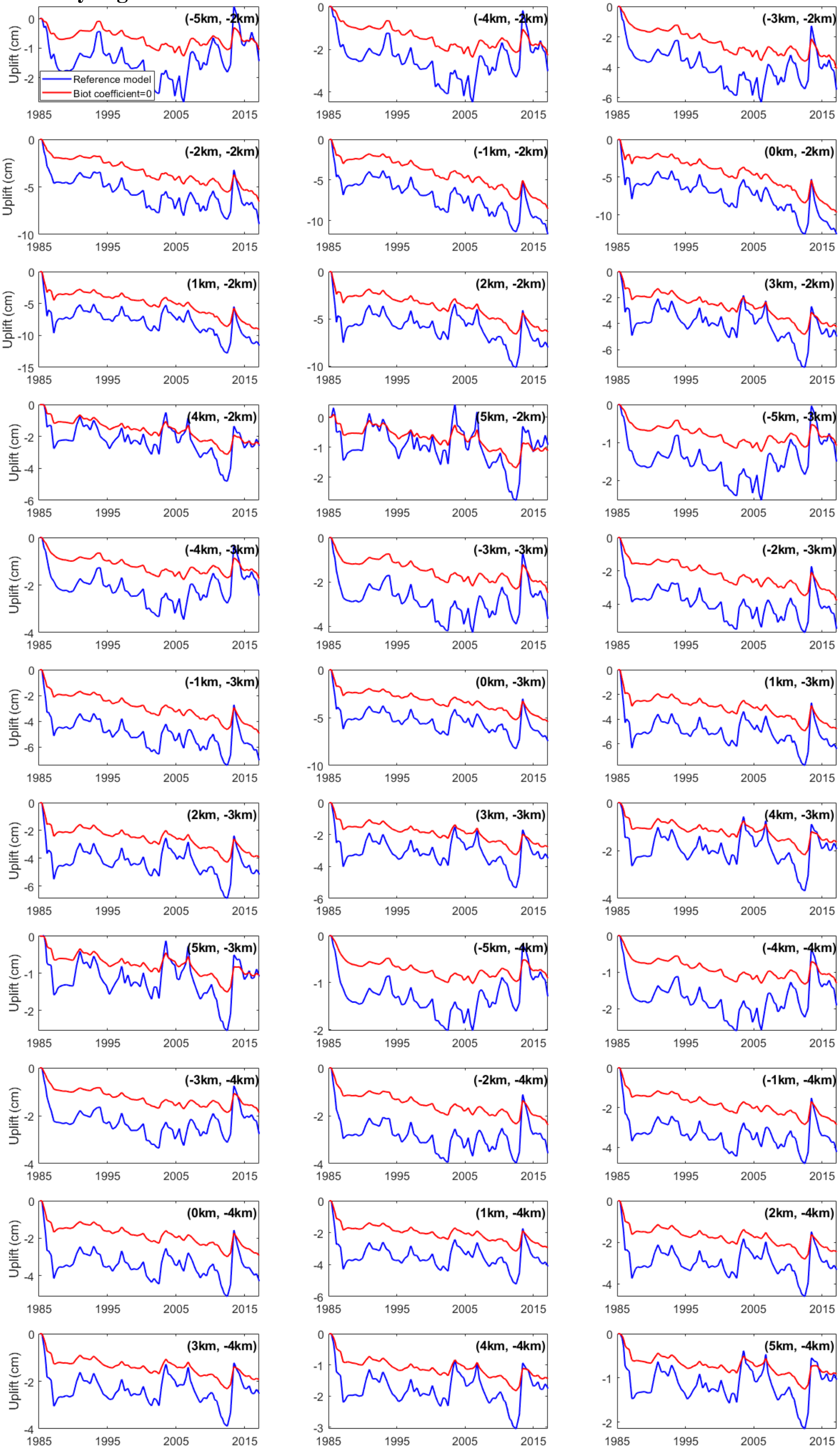


**Supplementary Fig. 18. Continued.**

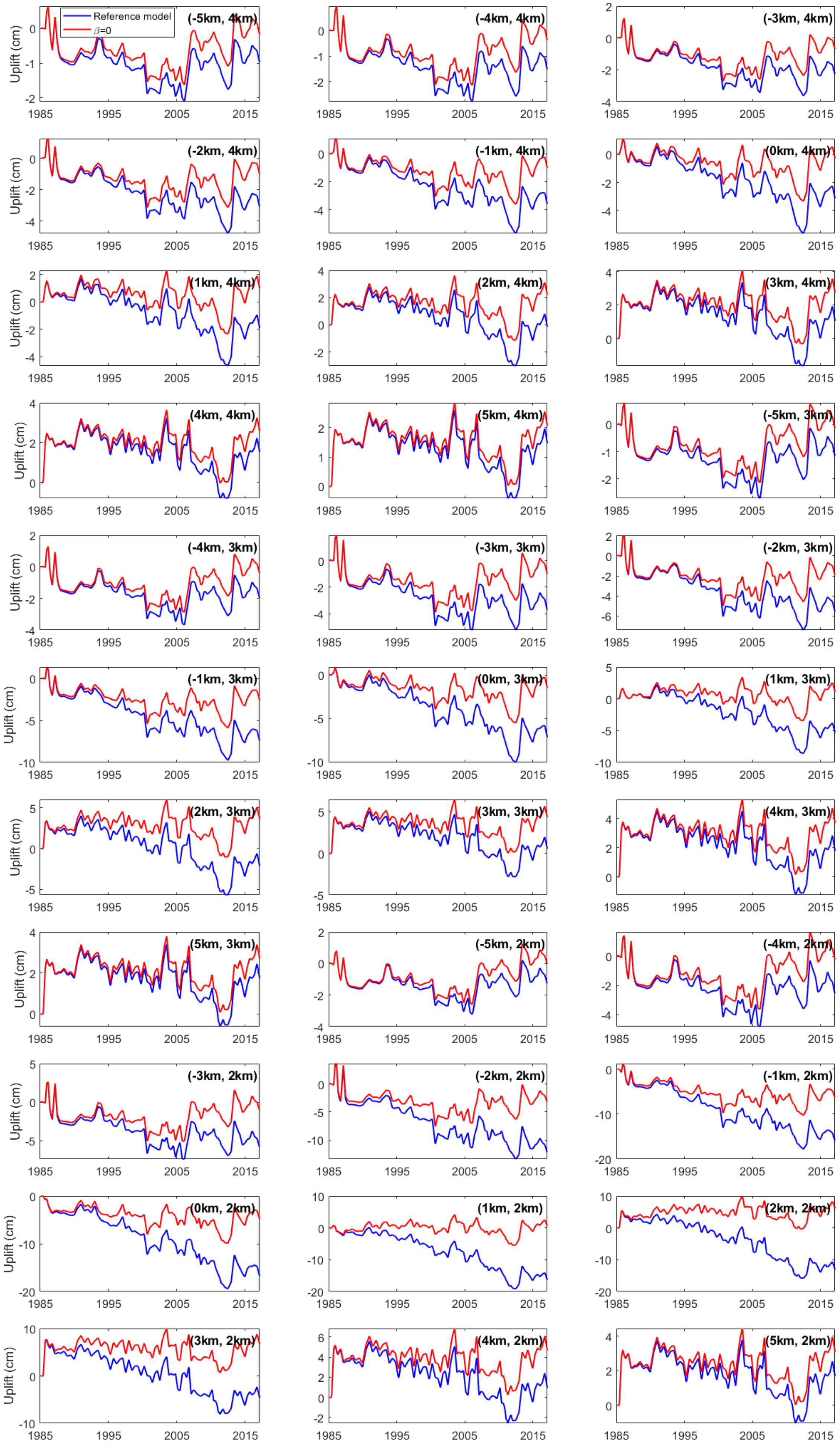

Reference model
β=0
Uplift (cm)
1985
1995
2005
2015
(-5km, 4km)
(-4km, 4km)
(-3km, 4km)
(-2km, 4km)
(-1km, 4km)
(0km, 4km)
(1km, 4km)
(2km, 4km)
(3km, 4km)
(4km, 4km)
(5km, 4km)
(-5km, 3km)
(-4km, 3km)
(-3km, 3km)
(-2km, 3km)
(-1km, 3km)
(0km, 3km)
(1km, 3km)
(2km, 3km)
(3km, 3km)
(4km, 3km)
(5km, 3km)
(-5km, 2km)
(-4km, 2km)
(-3km, 2km)
(-2km, 2km)
(-1km, 2km)
(0km, 2km)
(1km, 2km)
(2km, 2km)
(3km, 2km)
(4km, 2km)
(5km, 2km)

**Supplementary Fig. 19. Vertical surface displacement at 99 points simulated with the calibrated (reference) model and the model with the thermal expansion coefficients ($\beta$) of the two reservoirs and the feeder fault equal to 0.**

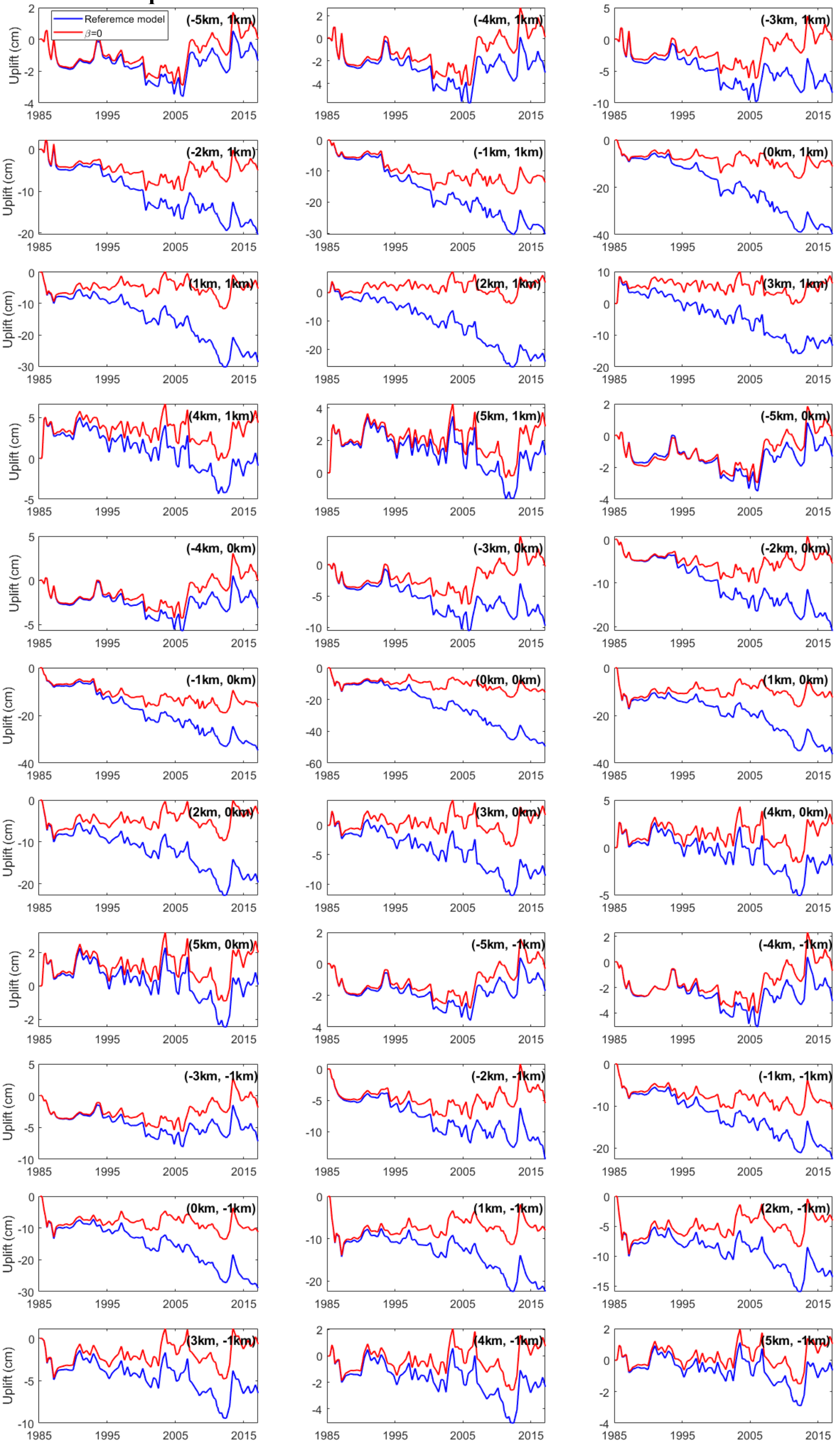

**Supplementary Fig. 19. Continued.**

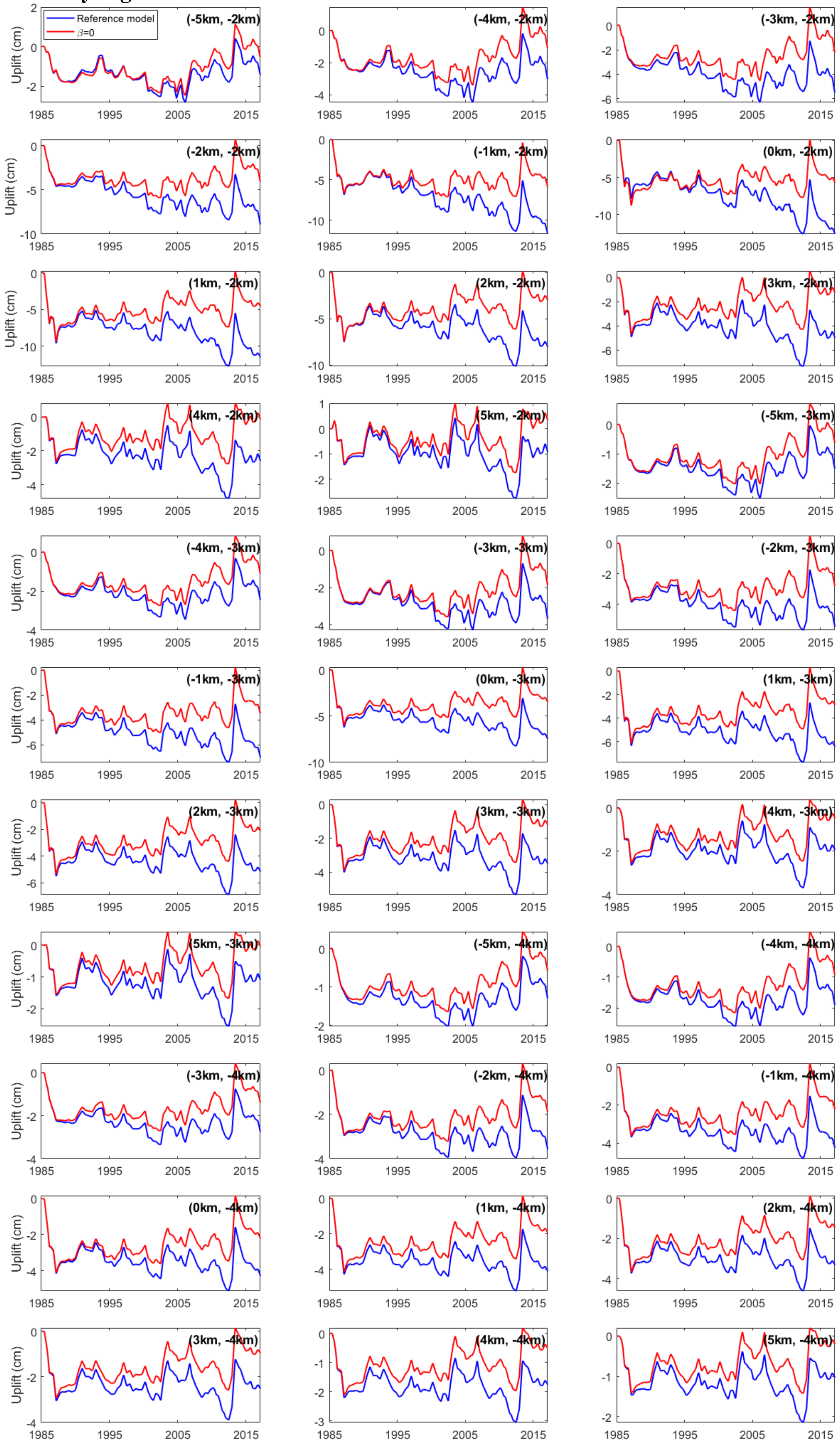


**Supplementary Fig. 19. Continued.**

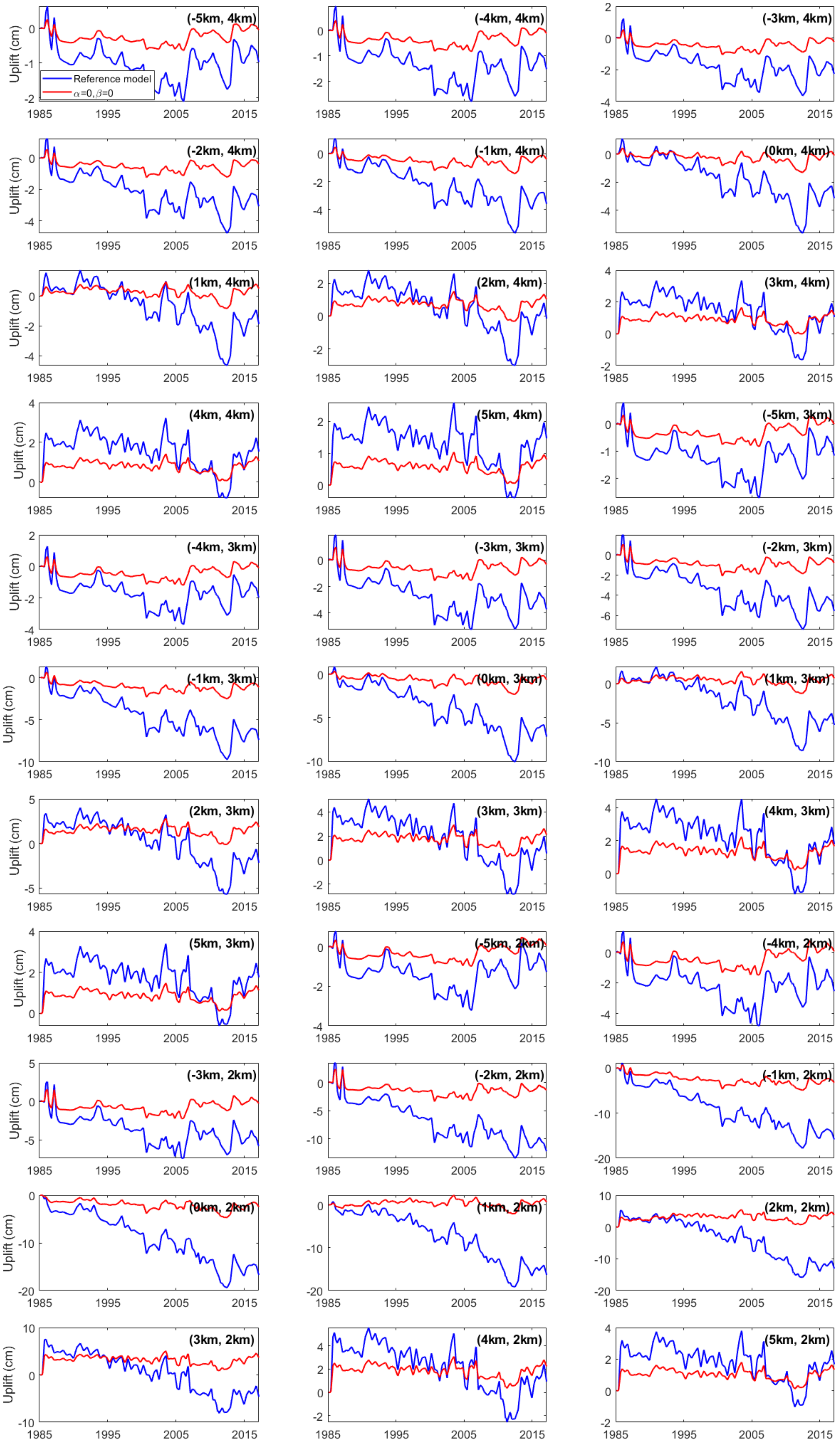
Reference model
α=0,β=0
Uplift (cm)
1985
1995
2005
2015
(-5km, 4km)
(-4km, 4km)
(-3km, 4km)
(-2km, 4km)
(-1km, 4km)
(0km, 4km)
(1km, 4km)
(2km, 4km)
(3km, 4km)
(4km, 4km)
(5km, 4km)
(-5km, 3km)
(-4km, 3km)
(-3km, 3km)
(-2km, 3km)
(-1km, 3km)
(0km, 3km)
(1km, 3km)
(2km, 3km)
(3km, 3km)
(4km, 3km)
(5km, 3km)
(-5km, 2km)
(-4km, 2km)
(-3km, 2km)
(-2km, 2km)
(-1km, 2km)
(0km, 2km)
(1km, 2km)
(2km, 2km)
(3km, 2km)
(4km, 2km)
(5km, 2km)

**Supplementary Fig. 20. Vertical surface displacement at 99 points simulated with the calibrated (reference) model and the model with the Biot ($\alpha$) and thermal expansion coefficients of the two reservoirs and the feeder fault equal to 0.**

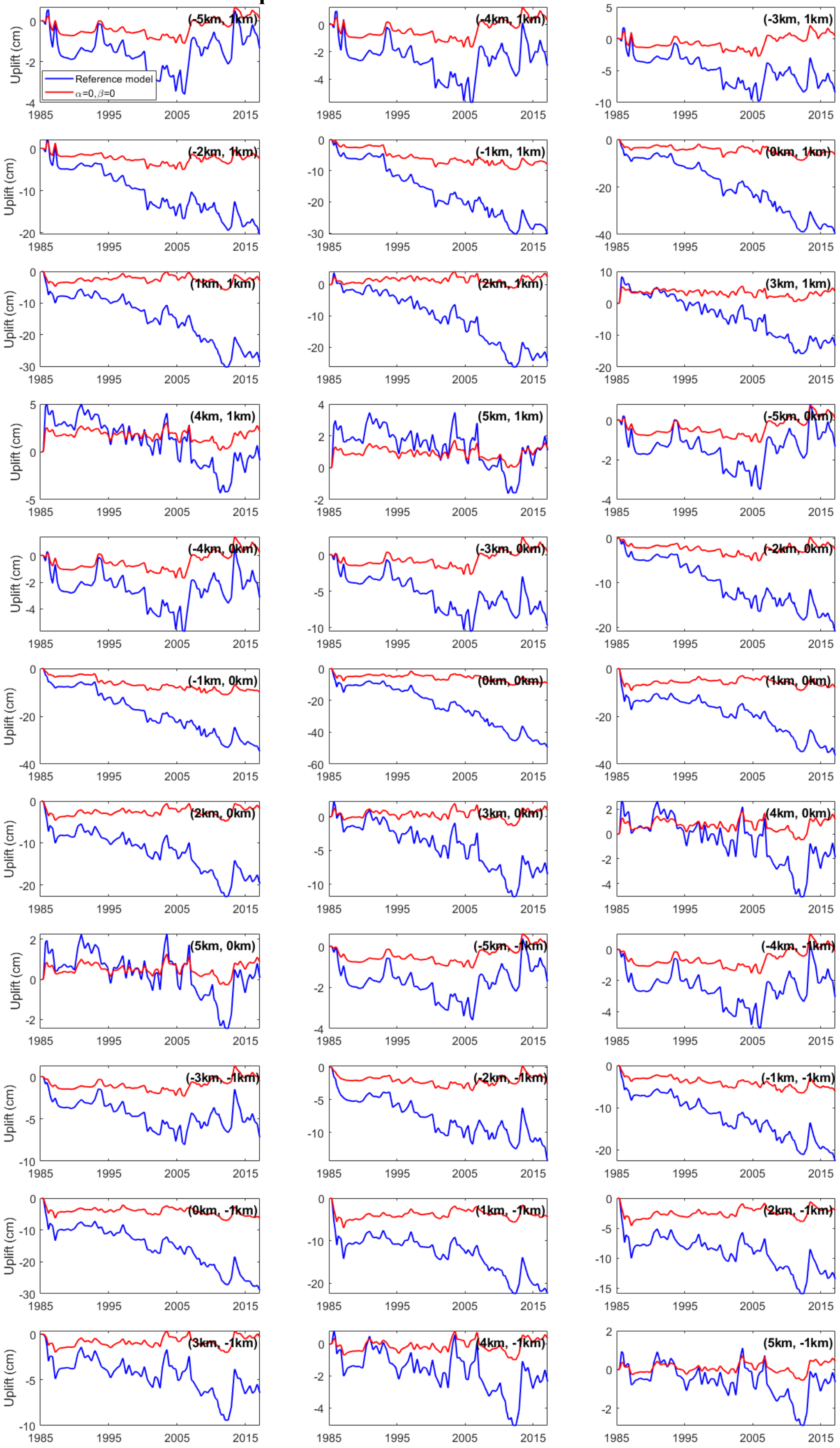

**Supplementary Fig. 20. Continued.**

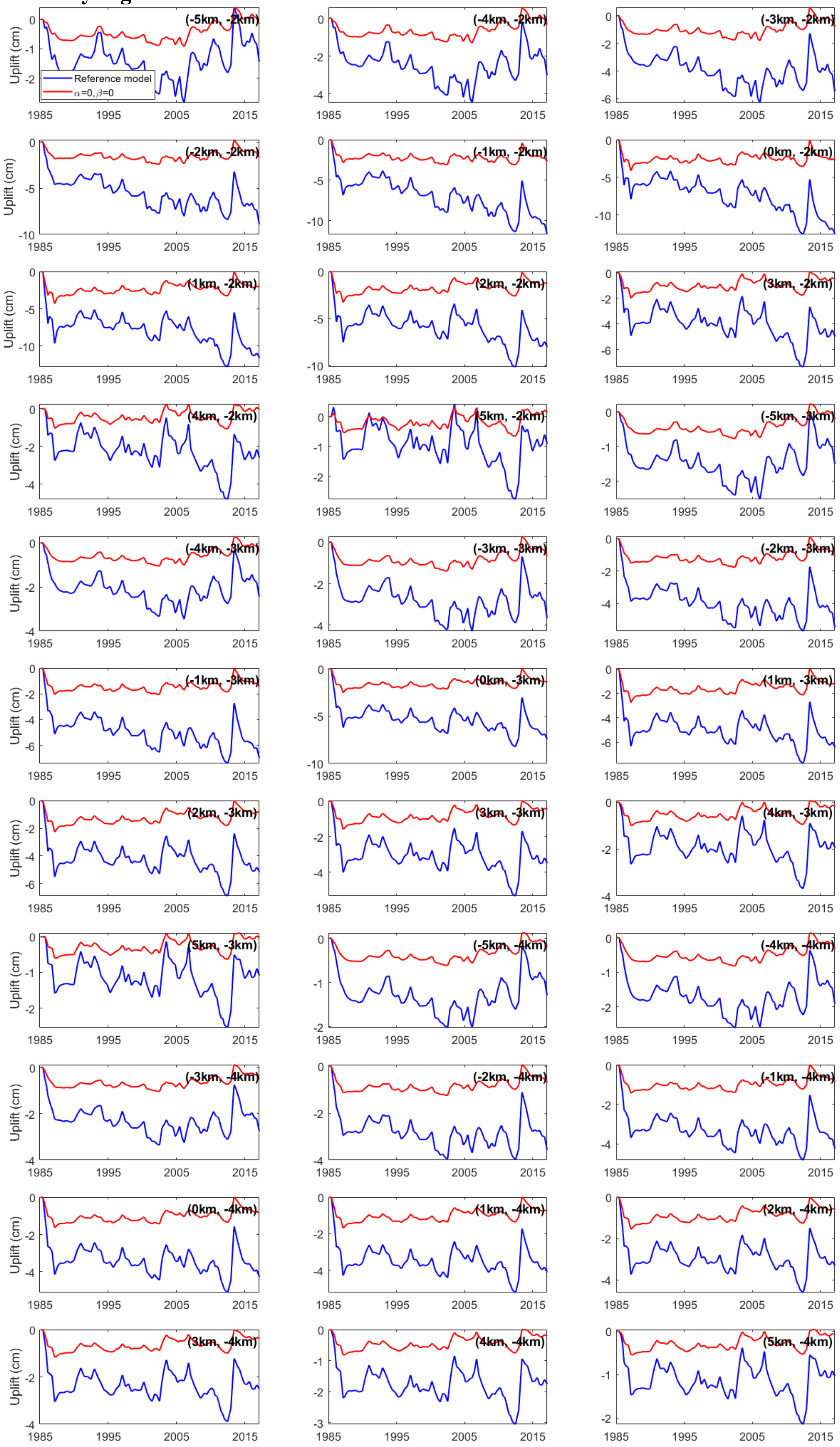


**Supplementary Fig. 20. Continued.**

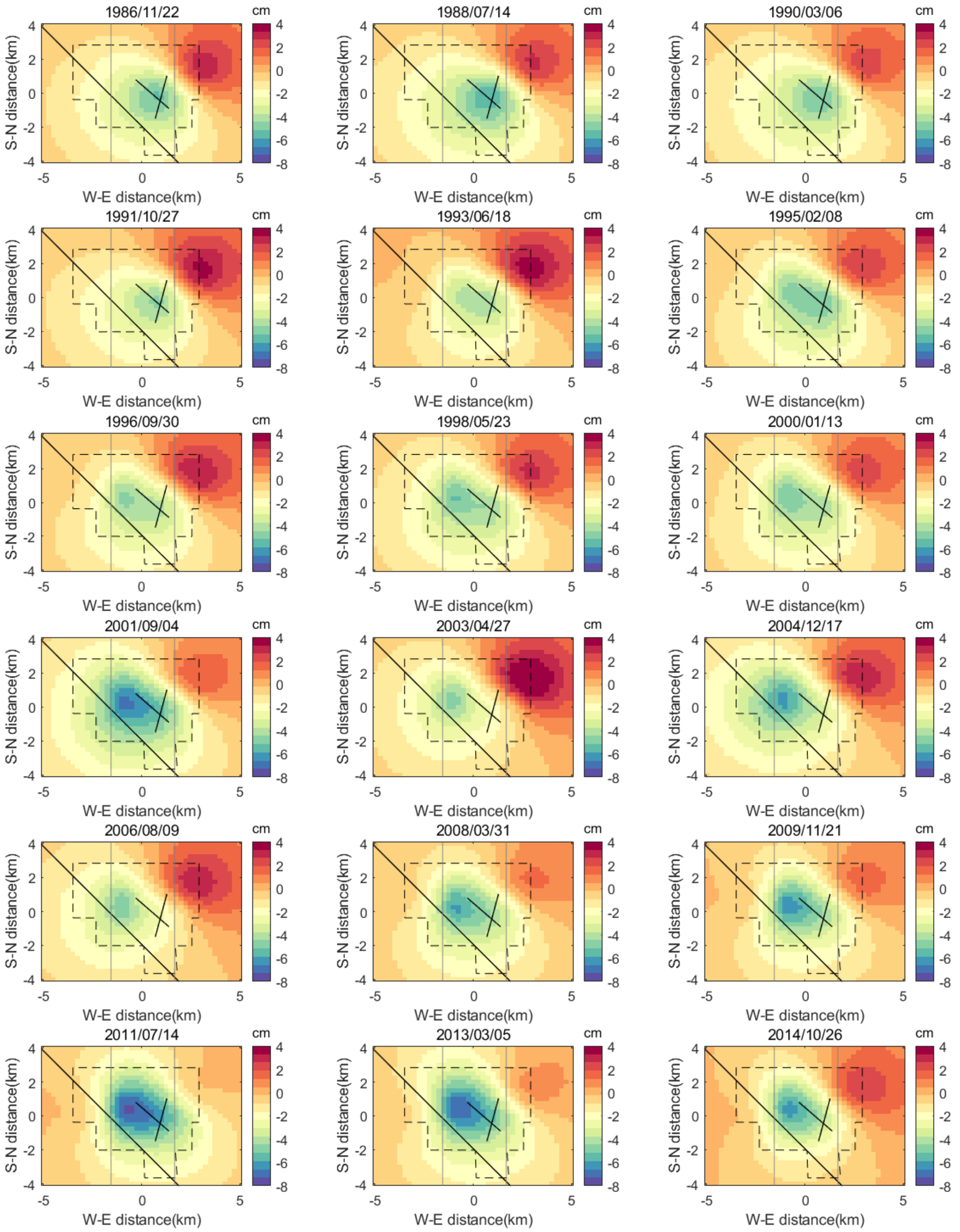


**Supplementary Fig. 21. Vertical surface displacement caused by pressure variation at different time nodes.**

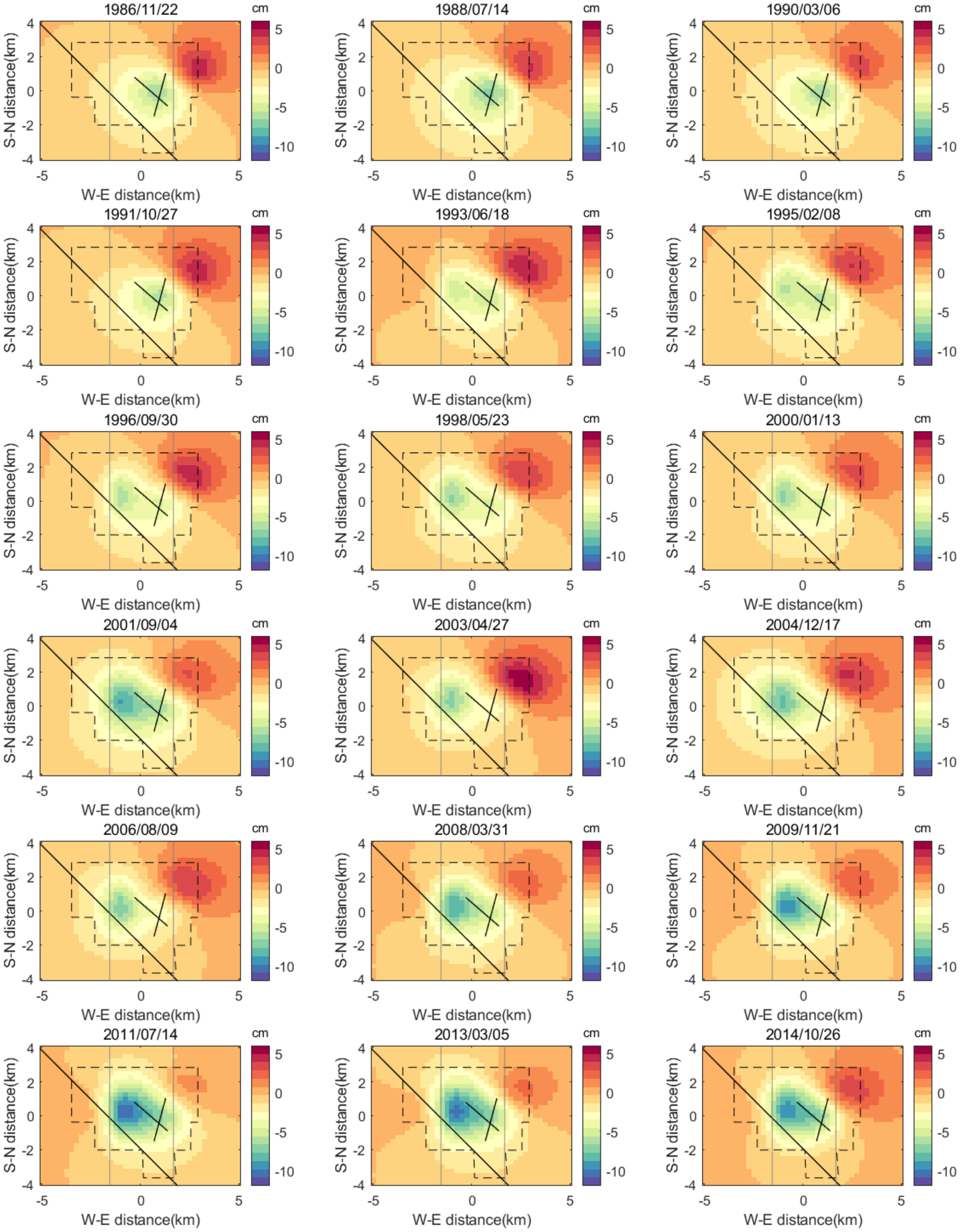


**Supplementary Fig. 22. Vertical surface displacement caused by poroelastic effects of expansion and contraction at different time nodes.**

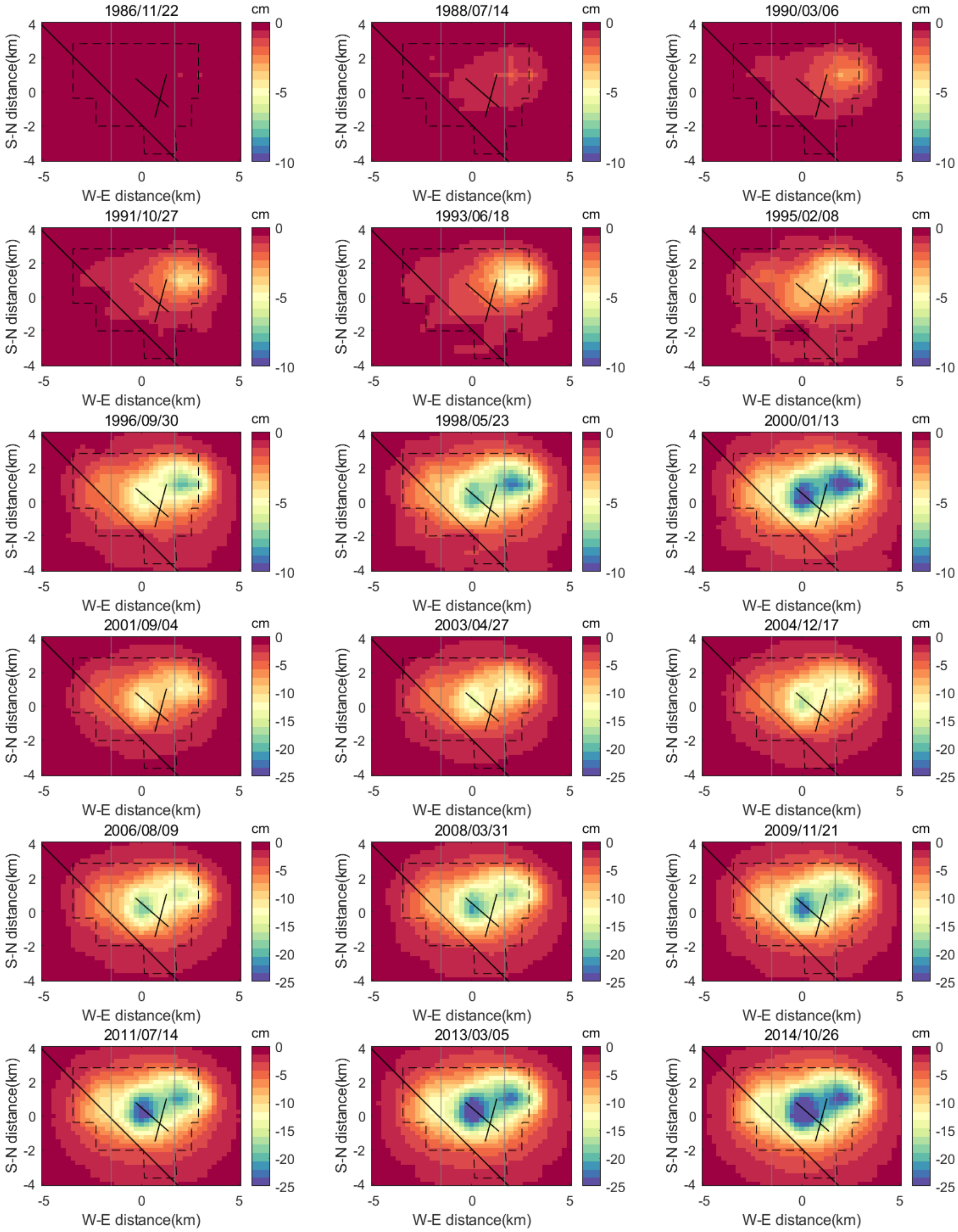


**Supplementary Fig. 23. Vertical surface displacement caused by thermoelastic effect of contraction at different time nodes.** As the thermal effect increases with time, we use two color scales for two time periods of1985-2000 and 2001-2014.

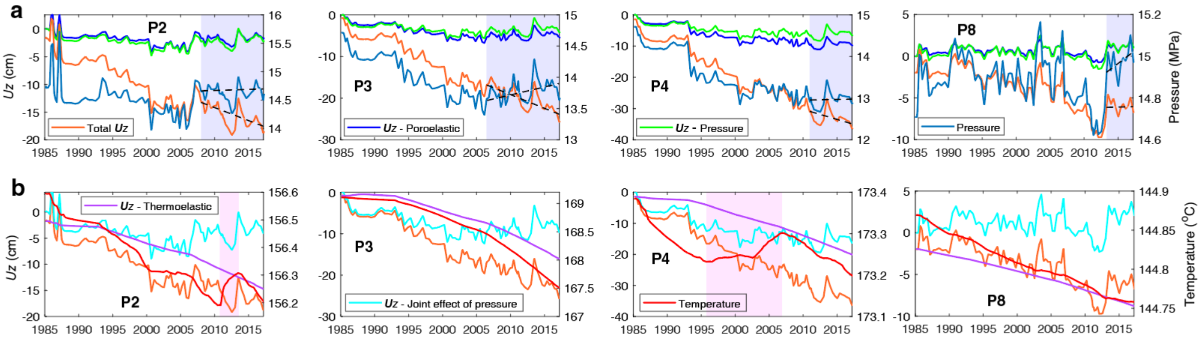


**Supplementary Fig. 24. Temporal evolution of thermo-poro-elastic effects at four probe points (see Figs. 7 and 8 in the main text for their locations). a** Temporal evolution of the effects of pressure fluctuation and poroelastic response at four probe points. Shaded regions mark the time periods with contrary trends of cumulative vertical displacement and pressure changes (marked with black dashed lines). **b** Temporal evolution of the thermal contraction effect at four probe points. Shaded regions show the time periods with opposing trends in cumulative vertical displacement and temperature.

## Supplementary tables

**Supplementary Table 1. 69 operation wells of the HGF.**

| Well ID | Injection time period | Extraction time period | Vertical well | Depth ranges of open-hole section (m) | Zone |
|---|---|---|---|---|---|
| 2590007 | 1993/06/01-2017/05/31 | | YES | 649.83-1072.9; 1190.55-1507.24 | West |
| 2590039 | 1993/06/01-2017/05/31 | | YES | 615.09-1584.96 | Central |
| 2590067 | 1985/05/01-2017/05/31 | | YES | 617.3-1173.3 | East |
| 2590536 | | 1985/04/01-2017/05/31 | NO | 1350.3-2280.5 | Central |
| 2590537 | | 1985/04/01-2017/05/31 | NO | 784.9-1334.4 | Central |
| 2590538 | | 1985/04/01-2013/08/10 | NO | 1282-1889.8 | Central |
| 2590539 | | 1985/10/01-2017/05/10 | NO | 777.2-1103.1 | Central |
| 2590540 | | 1985/05/01-2016/11/30 | NO | 865.6-1037.5; 2440.2-3211.4 | Central |
| 2590574 | | 1985/04/01-2017/04/09 | NO | 1325.9-2211.9 | Central |
| 2590575 | 2000/09/01-2017/05/31 | 1985/04/01-1989/03/01; 2001/10/01-2002/02/10 | NO | 2442.06-3269.59 | Central |
| 2590578 | | 1985/05/01-2017/05/31 | YES | 885.1-1495.5 | Central |
| 2590582 | | 1985/04/01-2017/05/31 | NO | 1050.3-1659.3 | Central |
| 2590583 | | 1985/05/01-2013/03/15 | NO | 799.5-1528.0 | Central |
| 2590584 | 2000/08/03-2017/05/31 | 1985/05/01-1989/03/01 | NO | 1213.1-2057.7 | Central |
| 2590585 | 2005/04/01-2017/05/31 | 1985/04/01-1989/03/01 | NO | 854-1400.1 | Central |
| 2590587 | 2001/01/01-2015/04/19 | 1985/06/01-1989/02/01 | NO | 1320.6-1994.1 | Central |
| 2590588 | 1993/06/01-2017/05/31 | 1985/04/01-1989/03/01 | NO | 886.3-1462.8 | Central |
| 2590589 | | 1985/05/01-2017/05/31 | NO | 1266.4-2081.5 | Central |
| 2590590 | 1985/06/01-2016/11/30 | | NO | 610.5-906.8; 1219.2-3028 | East |
| 2590591 | 2000/09/01-2017/05/31 | 1985/06/01-1989/03/01 | NO | 1302.6-1918.4 | Central |
| 2590592 | 2006/09/01-2017/05/31 | 1985/06/01-1989/03/01 | NO | 727.4-1528.4 | Central |
| 2590600 | 1985/05/01-2017/05/31 | | NO | 622.5-1349.3 | East |
| 2590601 | 1985/06/01-2017/05/31 | | NO | 1236.5-1950.8 | East |
| 2590602 | 1985/05/01-2010/04/05 | | NO | 654.5-1412.2 | West |
| 2590603 | 1985/04/01-2017/05/31 | | NO | 1255.1-2006.9 | West |
| 2590610 | 1985/06/01-2017/05/31 | | NO | 1257.8-2011.8 | East |
| 2590611 | 1985/06/01-2017/05/31 | | NO | 635.3-1307.2 | East |

| | | | | | |
|---|---|---|---|---|---|
| 2590612 | 1985/06/01-2017/05/31 | | NO | 1257.8-1895.9 | East |
| 2590613 | 1985/06/01-2017/05/31 | | NO | 642-1390.8 | East |
| 2590614 | 1985/04/01-2017/05/31 | | NO | 610.6-1336.5 | West |
| 2590615 | 1985/06/01-2017/05/31 | | NO | 1255.1-2006.9 | West |
| 2590616 | 1985/06/01-2009/06/05 | | NO | 625.2-1395.7 | West |
| 2590656 | 2000/09/01-2017/05/31 | 1987/02/01-1989/03/01 | NO | 2154.94-2598.42 | Central |
| 2590657 | 1987/01/01-2017/05/30 | | NO | 1261.87-2031.49 | West |
| 2590659 | 1986/12/01-2017/01/25 | | NO | 1269.19-2069.59 | West |
| 2590664 | 2000/08/01-2017/05/31 | | NO | 1973.58-2741.68 | Central |
| 2590666 | 1987/03/01-2017/05/31 | | NO | 1154.58-1854.71 | East |
| 2590667 | 1989/03/01-2017/05/31 | | NO | 1158.24-1696.21 | West |
| 2590668 | | 1987/06/01-2013/11/01 | NO | 694.6-1533.8 | Central |
| 2590680 | 1998/08/01-2017/05/31 | 1987/08/01-1988/01/18 | NO | 2202.5-2940 | Central |
| 2591211 | | 1993/06/01-2017/05/31 | YES | 751-1211 | Central |
| 2591212 | 2016/01/01-2017/01/31 | 1993/06/01-2015/04/20 | NO | 749.6-1230 | Central |
| 2591213 | | 1993/06/01-2017/05/31 | YES | 745.6-1226.3 | Central |
| 2591214 | | 1993/06/01-2017/05/31 | YES | 740.5-1825.7 | Central |
| 2591215 | | 1993/06/01-2017/05/31 | YES | 607.7-1228 | Central |
| 2591216 | | 1993/06/01-2017/05/31 | YES | 725-1220 | Central |
| 2591217 | | 1993/06/01-2017/05/31 | YES | 647-1231 | Central |
| 2591218 | | 1993/06/01-2017/05/31 | YES | 795-1843 | Central |
| 2591219 | | 1993/06/01-2017/05/29 | YES | 597.4-1220 | Central |
| 2591220 | | 1993/06/01-2017/05/31 | YES | 771-1211.8 | Central |
| 2591221 | | 1993/06/01-2017/05/31 | YES | 677.5-1236 | Central |
| 2591222 | 1993/06/01-2017/05/31 | | YES | 701.3-1371 | Central |
| 2591224 | 1993/06/01-2017/05/31 | | YES | 785.7-1370 | Central |
| 2591229 | 1993/05/01-2017/05/31 | | NO | 754.6-1295.4 | East |
| 2591265 | | 2006/01/01-2017/05/31 | YES | 773.5-1689.6 | Central |
| 2591424 | 2006/06/01-2017/05/29 | | NO | 827.53-2007.11 | East |
| 2591425 | 2008/04/01-2016/12/30 | | YES | 1302.11-1876 | Central |
| 2591430 | | 2007/01/01-2017/05/31 | YES | 548.6-754.7 | Central |
| 2591431 | 2006/10/01-2017/05/31 | | NO | 643.74-1935.18 | West |

| | | | | | |
|---|---|---|---|---|---|
| 2591432 | 2008/04/01-2017/05/31 | | YES | 775.41-1823.31 | Central |
| 2591435 | | 2008/04/01-2017/05/31 | YES | 860.5-1212.5 | Central |
| 2591439 | 2008/04/01-2017/05/31 | | NO | 964.08-1774.55 | Central |
| 2591441 | | 2008/04/01-2009/07/05 | NO | 844-924.5 | Central |
| 2591478 | | 2009/07/01-2017/05/31 | YES | 705.6-1529.2 | Central |
| 2591489 | | 2011/01/01-2017/05/31 | YES | 711.7-1467.9 | Central |
| 2591497 | | 2011/06/01-2017/05/31 | YES | 569.4-991.2 | Central |
| 2591506 | | 2013/05/01-2017/05/31 | YES | 581.3-1004.9 | Central |
| 2591507 | | 2013/05/01-2017/05/31 | YES | 587-1195 | Central |
| 2591511 | | 2016/01/01-2017/05/18 | NO | 564.8-1081.1 | Central |

**Supplementary Table 2. Mechanical, hydraulic and thermophysical parameters in the Heber geothermal reservoir model**

| Formation | Thickness (km) | Density (kg/m$^3$) | Young's modulus (GPa) | Biot coefficient | | Poisson's ratio | Porosity (%) | Permeability ($10^{-15}$ m$^2$) | | Specific heat (J/(kg·°C)[c] | Thermal conductivity (W/(m·°C)) | Thermal expansion coefficient (°C$^{-1}$) |
|---|---|---|---|---|---|---|---|---|---|---|---|---|
| | | | | I[a] | II[b] | | | Horizontal | Vertical | | | |
| Caprock | 0.55 | 1719 | 0.31 | 0.97 | 0.98 | 0.49 | 3 | 0.28 | 0.28 | 1000 | 1 | 1E-5 |
| Upper reservoir | 1.10 | 2045 | 5.24 | **0.85** | 0.91 | 0.42 | **18** | **20** | **20** | **1000** | **2→3** | **1E-5 →1.3E-5** |
| Lower reservoir | 1.50 | 2362 | 27.87 | **0.67** | 0.76 | 0.28 | **18** | **20→10** | **20→10** | **1000** | **2→3** | **1E-5 →1.3E-5** |
| Basal layer | 1.15 | 2565 | 60.33 | 0.57 | 0.44 | 0.22 | 10 | 5 | 0.28 | 1000 | 2 | 1E-5 |
| Basement | 2.70 | 2622 | 69.15 | 0.56 | 0.39 | 0.23 | 5 | 1 | 0.28 | 1000 | 1 | 1E-5 |
| Feeder fault | **0.30** | 2500 | **10** | 0.70 | 0.85 | **0.3** | **20** | **20** | **20** | **1000** | **2** | **1E-5** |
| Boundary fault | **0.10** | 2500 | 10 | 0.64 | 0.85 | 0.3 | **5** | **5** | **5** | **1000** | **1** | 1E-5 |
| Subsidiary fault | **0.10** | 2500 | 10 | 0.70 | 0.85 | 0.3 | **20** | **20** | **20** | **1000** | **1** | 1E-5 |

[a]The Biot values are calculated with the equation: $\alpha = (1 + \nu + 2\phi(1 - 2\nu))/(3(1 - \nu))$ (*Zimmerman et al., 1986*). $\nu$ and $\phi$ are the Poisson's ratio and porosity of rock within each formation.

[b]The Biot values are calculated with the equation: $\alpha = 1 - K/K_s'$ (*Wang,* 2000), where the drained bulk modulus $K$ is equal to $E/[3(1 - 2\nu)]$. $K_s'$ is the unjacketed bulk modulus, often called the solid-grain modulus, and is assumed to be 100 GPa.

[c]The value of specific heat varies from 800 J/(kg·°C) to 1200 J/(kg·°C) with the temperature increasing from 50°C to 200°C for different rocks (*Roberston, 1988*).

Yellow cells mark the parameters considered in model calibration. The cells with one value indicate that the initial parameter setting is optimal. For the cells with two values, the values at the start and end of each arrow correspond to the initial parameter setting and the calibration result, respectively.

**Supplementary Table 3. Misfits between simulation results with different model sizes and observations.**

| Model size | | | RMS misfit | | |
|---|---|---|---|---|---|
| Length (km) | Width (km) | Height (km) | Leveling (cm) | InSAR (cm) | Temperature (°C) |
| 15 | 13 | 7 | 3.99 | 2.44 | 21.51 |
| 15 | 13 | 8 | 3.59 | 2.77 | 21.84 |
| 16 | 14 | 8 | 2.90 | 2.86 | 21.55 |
| 17 | 15 | 8 | 2.96 | 2.63 | 21.42 |
| 17 | 15 | 9 | 3.26 | 2.90 | 21.69 |

**Supplementary Table 4. Misfits of model predictions with different reservoir porosities to surface displacement and temperature observations. Orange and green values correspond to the first and second rounds of model calibration, respectively.**

**I. RMS misfits to Leveling observations (cm)**

| Porosity | | Upper reservoir | | | |
|---|---|---|---|---|---|
| | | 14% | 16% | 18% | 20% |
| Lower reservoir | 14% | 3.17 | 3.33 | 3.13 | 3.38 |
| | 16% | 3.30 | 2.98 / 2.48 | 3.21 / 2.51 | 3.27 / 3.28 |
| | 18% | 3.72 | 3.10 / 2.70 | 2.90 / 2.39 | 3.43 / 3.35 |
| | 20% | 3.51 | 3.44 / 3.18 | 3.02 / 2.80 | 3.56 / 2.80 |

**II. RMS misfits to InSAR observations (cm)**

| Porosity | | Upper reservoir | | | |
|---|---|---|---|---|---|
| | | 14% | 16% | 18% | 20% |
| Lower reservoir | 14% | 2.62 | 2.71 | 2.38 | 2.54 |
| | 16% | 2.48 | 2.69 / 2.61 | 2.69 / 2.48 | 2.62 / 5.04 |
| | 18% | 3.47 | 2.68 / 3.43 | 2.86 / 2.68 | 2.52 / 3.02 |
| | 20% | 2.87 | 3.04 / 2.63 | 2.68 / 2.81 | 2.63 / 2.47 |

**III. Weighted RMS misfits of deformation[a] (cm)**

| Porosity | | Upper reservoir | | | |
|---|---|---|---|---|---|
| | | 14% | 16% | 18% | 20% |
| Lower reservoir | 14% | 3.06 | 3.21 | 2.98 | 3.21 |
| | 16% | 3.14 | 2.92 / 2.51 | 3.11 / 2.50 | 3.14 / 3.63 |
| | 18% | 3.67 | 3.02 / 2.85 | 2.89 / 2.45 | 3.25 / 3.28 |
| | 20% | 3.38 | 3.36 / 3.07 | 2.95 / 2.80 | 3.37 / 2.73 |

**IV. RMS misfits to well temperature (°C)**

| Porosity | | Upper reservoir | | | |
|---|---|---|---|---|---|
| | | 14% | 16% | 18% | 20% |
| Lower reservoir | 14% | 21.25 | 22.51 | 21.67 | 21.15 |
| | 16% | 21.19 | 21.15 / 21.71 | 21.48 / 20.61 | 20.79 / 21.72 |
| | 18% | 20.95 | 21.16 / 20.84 | 21.55 / 20.30 | 20.71 / 20.61 |
| | 20% | 21.40 | 21.59 / 20.57 | 20.96 / 20.26 | 20.81 / 20.90 |

**[a]The weighted misfits are calculated with the equation: 0.8×(Leveling misfits) + 0.2×(InSAR misfits)**

**Supplementary Table 5. Misfits of model predictions with different permeabilities of the upper reservoir to surface displacement and temperature observations. Orange and green values correspond to the first and second rounds of model calibration, respectively.**

**I. RMS misfits to Leveling observations (cm)**

| Permeability | | Horizontal | | |
|---|---|---|---|---|
| | | 10 mD | 20 mD | 30 mD |
| Vertical | 10 mD | 3.52<br>2.87 | 3.21<br>2.47 | 3.42<br>2.81 |
| | 20 mD | 3.38<br>3.63 | 2.90<br>2.39 | 3.10<br>2.89 |
| | 30 mD | 3.03<br>2.91 | 3.19<br>2.44 | 3.40<br>3.11 |

**II. RMS misfits to InSAR observations (cm)**

| Permeability | | Horizontal | | |
|---|---|---|---|---|
| | | 10 mD | 20 mD | 30 mD |
| Vertical | 10 mD | 2.55<br>2.98 | 2.61<br>2.50 | 2.78<br>2.52 |
| | 20 mD | 4.19<br>2.83 | 2.86<br>2.68 | 2.58<br>2.54 |
| | 30 mD | 2.99<br>2.78 | 2.73<br>3.46 | 2.75<br>2.87 |

**III. Weighted RMS misfits of deformation (cm)**

| Permeability | | Horizontal | | |
|---|---|---|---|---|
| | | 10 mD | 20 mD | 30 mD |
| Vertical | 10 mD | 3.33<br>2.89 | 3.09<br>3.48 | 3.29<br>2.75 |
| | 20 mD | 3.54<br>3.47 | 2.89<br>2.45 | 3.00<br>2.82 |
| | 30 mD | 3.02<br>2.88 | 3.10<br>2.64 | 3.27<br>3.06 |

**IV. RMS misfits to well temperature (°C)**

| Permeability | | Horizontal | | |
|---|---|---|---|---|
| | | 10 mD | 20 mD | 30 mD |
| Vertical | 10 mD | 21.23<br>21.43 | 21.09<br>20.12 | 20.93<br>20.47 |
| | 20 mD | 20.60<br>20.86 | 21.55<br>20.30 | 21.26<br>20.63 |
| | 30 mD | 20.79<br>20.89 | 20.45<br>21.29 | 21.11<br>20.97 |

**Supplementary Table 6. Misfits of model predictions with different permeabilities of the lower reservoir to surface displacement and temperature observations. Orange and green values correspond to the first and second rounds of model calibration, respectively.**

**I. RMS misfits to Leveling observations (cm)**

| Permeability | | Horizontal | | |
|---|---|---|---|---|
| | | 5 mD | 10 mD | 20 mD |
| Vertical | 5 mD | 2.88<br>2.51 | 3.62<br>3.22 | 2.91<br>2.73 |
| | 10 mD | 3.05<br>2.71 | 2.70<br>2.39 | 3.39<br>2.74 |
| | 20 mD | 3.17<br>2.95 | 4.09<br>3.40 | 2.90<br>2.68 |

**II. RMS misfits to InSAR observations (cm)**

| Permeability | | Horizontal | | |
|---|---|---|---|---|
| | | 5 mD | 10 mD | 20 mD |
| Vertical | 5 mD | 2.49<br>2.62 | 3.00<br>2.58 | 2.63<br>2.75 |
| | 10 mD | 3.55<br>4.35 | 2.63<br>2.68 | 2.45<br>2.68 |
| | 20 mD | 3.35<br>4.01 | 2.81<br>2.90 | 2.86<br>2.67 |

**III. Weighted RMS misfits of deformation (cm)**

| Permeability | | Horizontal | | |
|---|---|---|---|---|
| | | 5 mD | 10 mD | 20 mD |
| Vertical | 5 mD | 2.80<br>2.53 | 3.50<br>3.09 | 2.85<br>2.73 |
| | 10 mD | 3.15<br>3.04 | 2.69<br>2.45 | 3.20<br>2.73 |
| | 20 mD | 3.21<br>3.16 | 3.83<br>3.30 | 2.89<br>2.68 |

**IV. RMS misfits to well temperature (°C)**

| Permeability | | Horizontal | | |
|---|---|---|---|---|
| | | 5 mD | 10 mD | 20 mD |
| Vertical | 5 mD | 20.72<br>20.69 | 21.59<br>21.48 | 20.93<br>20.93 |
| | 10 mD | 21.09<br>21.09 | 20.27<br>20.30 | 20.64<br>20.84 |
| | 20 mD | 21.11<br>21.25 | 21.12<br>20.95 | 21.55<br>21.85 |

**Supplementary Table 7. Misfits of model predictions with different reservoir Biot coefficients to surface displacement and temperature observations. Orange and green values correspond to the first and second rounds of model calibration, respectively.**

**I. RMS misfits to Leveling observations (cm)**

| Biot coefficient | | Upper reservoir | | |
|---|---|---|---|---|
| | | 0.7 | 0.85 | 1 |
| Lower reservoir | 0.52 | 2.77 | 2.73 | 2.69 |
| | 0.67 | 2.76 | 2.70 | 2.69 |
| | 0.82 | 2.76 | 2.72 | 2.69 |

**II. RMS misfits to InSAR observations (cm)**

| Biot coefficient | | Upper reservoir | | |
|---|---|---|---|---|
| | | 0.7 | 0.85 | 1 |
| Lower reservoir | 0.52 | 2.55 | 2.60 | 2.66 |
| | 0.67 | 2.58 | 2.63 | 2.69 |
| | 0.82 | 2.61 | 2.67 | 2.73 |

**III. Weighted RMS misfits of deformation (cm)**

| Biot coefficient | | Upper reservoir | | |
|---|---|---|---|---|
| | | 0.7 | 0.85 | 1 |
| Lower reservoir | 0.52 | 2.73 | 2.70 | 2.68 |
| | 0.67 | 2.72 | 2.69 | 2.69 |
| | 0.82 | 2.73 | 2.71 | 2.70 |

**IV. RMS misfits to well temperature (°C)**

| Biot coefficient | | Upper reservoir | | |
|---|---|---|---|---|
| | | 0.7 | 0.85 | 1 |
| Lower reservoir | 0.52 | 20.27 | 20.27 | 20.27 |
| | 0.67 | 20.27 | 20.27 | 20.27 |
| | 0.82 | 20.27 | 20.27 | 20.27 |

**Supplementary Table 8. Misfits of model predictions with different thermophysical properties of the two reservoir layers to surface displacement and temperature observations. Orange and green values correspond to the first and second rounds of model calibration, respectively.**

| RMS misfit | Thermal Expansion Coefficient (°C$^{-1}$) | | | | | | Thermal Conductivity (W/(m·°C)) | | | | |
|---|---|---|---|---|---|---|---|---|---|---|---|
| | $8\times10^{-6}$ | $1\times10^{-5}$ | $1.3\times10^{-5}$ | $1.5\times10^{-5}$ | $1.8\times10^{-5}$ | $2\times10^{-5}$ | 1 | 2 | 3 | 4 | 5 |
| Leveling (cm) | 3.01 | 2.70<br>2.65 | 2.50<br>2.39 | 2.54<br>2.39 | 2.85<br>2.66 | 3.16 | 2.50 | 2.50 | 2.39 | 2.39 | 2.52 |
| InSAR (cm) | 2.62 | 2.63<br>2.63 | 2.68<br>2.68 | 2.73<br>2.73 | 2.81<br>2.81 | 2.87 | 2.73 | 2.68 | 2.68 | 2.73 | 3.18 |
| Weighted Misfit (cm) | 2.93 | 2.69<br>2.65 | 2.54<br>2.45 | 2.58<br>2.46 | 2.84<br>2.69 | 3.10 | 2.55 | 2.54 | 2.45 | 2.46 | 2.65 |
| Temperature (°C) | 20.27 | 20.27<br>20.30 | 20.27<br>20.30 | 20.27<br>20.30 | 20.27<br>20.30 | 20.27 | 20.25 | 20.27 | 20.30 | 20.34 | 20.36 |
| RMS misfit | Specific Heat (J/(kg·°C)) | | | | | | | | | | |
| | 800 | 900 | 1000 | 1100 | 1200 | | | | | | |
| Leveling (cm) | 2.68 | 2.54 | 2.39 | 2.44 | 2.95 | | | | | | |
| InSAR (cm) | 3.99 | 2.78 | 2.68 | 2.69 | 2.47 | | | | | | |
| Weighted Misfit (cm) | 2.94 | 2.59 | 2.45 | 2.49 | 2.85 | | | | | | |
| Temperature (°C) | 20.24 | 20.88 | 20.30 | 20.80 | 20.48 | | | | | | |

**Supplementary Table 9. Misfits of model predictions with different thicknesses and properties (porosity, Young's modulus, Poisson's ratio, and three thermophysical parameters) of the normal-slip feeder fault to surface displacement and temperature observations.**

| RMS misfit | Thickness (m) | | | | Porosity (%) | | | | E (GPa) | | | Poisson's Ratio | | |
|---|---|---|---|---|---|---|---|---|---|---|---|---|---|---|
| | 250 | 300 | 350 | 400 | 18 | 20 | 22 | 24 | 5 | 10 | 20 | 0.2 | 0.3 | 0.4 |
| Leveling (cm) | 2.88 | 2.39 | 3.32 | 2.78 | 2.65 | 2.39 | 2.62 | 2.62 | 2.40 | 2.39 | 2.39 | 2.39 | 2.39 | 2.39 |
| InSAR (cm) | 3.05 | 2.68 | 2.48 | 2.78 | 3.89 | 2.68 | 2.52 | 2.55 | 2.69 | 2.68 | 2.68 | 2.68 | 2.68 | 2.68 |
| Weighted Misfit (cm) | 2.91 | 2.45 | 3.15 | 2.78 | 2.90 | 2.45 | 2.60 | 2.61 | 2.46 | 2.45 | 2.45 | 2.45 | 2.45 | 2.45 |
| Temperature (°C) | 21.09 | 20.30 | 20.93 | 20.99 | 20.43 | 20.30 | 20.71 | 20.63 | 20.30 | 20.30 | 20.30 | 20.30 | 20.30 | 20.30 |
| RMS misfit | Thermal Expansion Coefficient (°C$^{-1}$) | | | | Thermal Conductivity (W/(m·°C)) | | | | Specific Heat (J/(kg·°C)) | | | | | |
| | $8\times10^{-6}$ | $1\times10^{-5}$ | $1.5\times10^{-5}$ | $2\times10^{-5}$ | 1 | 2 | 3 | 4 | 900 | 1000 | 1100 | | | |
| Leveling (cm) | 2.39 | 2.39 | 2.39 | 2.38 | 2.50 | 2.39 | 2.40 | 2.60 | 2.50 | 2.39 | 2.41 | | | |
| InSAR (cm) | 2.68 | 2.68 | 2.69 | 2.69 | 2.57 | 2.68 | 3.18 | 2.47 | 2.57 | 2.68 | 2.72 | | | |
| Weighted Misfit (cm) | 2.45 | 2.45 | 2.45 | 2.45 | 2.51 | 2.45 | 2.56 | 2.57 | 2.51 | 2.45 | 2.47 | | | |
| Temperature (°C) | 20.30 | 20.30 | 20.30 | 20.30 | 20.27 | 20.30 | 20.28 | 20.37 | 20.35 | 20.30 | 20.38 | | | |

**Supplementary Table 10. Misfits of model predictions with different permeabilities of the normal-slip fault to surface displacement and temperature observations.**

**I. RMS misfits to Leveling observations (cm)**

| Permeability | | Horizontal | | |
|---|---|---|---|---|
| | | 10 mD | 20 mD | 30 mD |
| Vertical | 10 mD | 2.78 | 2.89 | 3.02 |
| | 20 mD | 3.37 | 2.39 | 2.64 |
| | 30 mD | 2.74 | 2.80 | 2.98 |

**II. RMS misfits to InSAR observations (cm)**

| Permeability | | Horizontal | | |
|---|---|---|---|---|
| | | 10 mD | 20 mD | 30 mD |
| Vertical | 10 mD | 2.74 | 4.14 | 2.50 |
| | 20 mD | 2.68 | 2.68 | 2.57 |
| | 30 mD | 3.57 | 2.53 | 2.75 |

**III. Weighted RMS misfits of deformation (cm)**

| Permeability | | Horizontal | | |
|---|---|---|---|---|
| | | 10 mD | 20 mD | 30 mD |
| Vertical | 10 mD | 2.77 | 3.14 | 2.92 |
| | 20 mD | 3.23 | 2.45 | 2.63 |
| | 30 mD | 2.91 | 2.75 | 2.93 |

**IV. RMS misfits to well temperature (°C)**

| Permeability | | Horizontal | | |
|---|---|---|---|---|
| | | 10 mD | 20 mD | 30 mD |
| Vertical | 10 mD | 20.69 | 21.04 | 21.03 |
| | 20 mD | 20.56 | 20.30 | 20.74 |
| | 30 mD | 23.03 | 20.89 | 20.33 |

**Supplementary Table 11. Misfits of model predictions with different thicknesses and properties (porosity, thermal conductivity and specific heat) of the plate boundary fault to surface displacement and temperature observations.**

| RMS misfit | Thickness (m) | | | Porosity (%) | | | |
|---|---|---|---|---|---|---|---|
| | 50 | 100 | 150 | 1 | 3 | 5 | 7 |
| Leveling (cm) | 3.03 | 2.39 | 3.15 | 2.72 | 2.59 | 2.39 | 2.56 |
| InSAR (cm) | 2.66 | 2.68 | 3.19 | 2.55 | 2.67 | 2.68 | 2.84 |
| Weighted Misfit (cm) | 2.96 | 2.45 | 3.16 | 2.69 | 2.61 | 2.45 | 2.62 |
| Temperature (°C) | 21.93 | 20.30 | 20.88 | 20.70 | 20.61 | 20.30 | 20.57 |
| **RMS misfit** | **Thermal Conductivity (W/(m·°C))** | | | | **Specific heat (J/(kg·°C))** | | |
| | 0.5 | 1 | 2 | 3 | 900 | 1000 | 1100 |
| Leveling (cm) | 2.39 | 2.39 | 2.39 | 2.39 | 2.39 | 2.39 | 2.39 |
| InSAR (cm) | 2.68 | 2.68 | 2.68 | 2.68 | 2.68 | 2.68 | 2.68 |
| Weighted Misfit (cm) | 2.45 | 2.45 | 2.45 | 2.45 | 2.45 | 2.45 | 2.45 |
| Temperature (°C) | 20.30 | 20.30 | 20.30 | 20.30 | 20.30 | 20.30 | 20.30 |

**Supplementary Table 12. Misfits of model predictions with different permeabilities of the plate boundary fault to surface displacement and temperature observations.**

**I. RMS misfits to Leveling observations (cm)**

| Permeability | | Horizontal | | |
|---|---|---|---|---|
| | | 1 mD | 5 mD | 10 mD |
| Vertical | 1 mD | 3.38 | 2.71 | 2.83 |
| | 5 mD | 2.97 | 2.39 | 3.05 |
| | 10 mD | 2.60 | 2.45 | 3.11 |

**II. RMS misfits to InSAR observations (cm)**

| Permeability | | Horizontal | | |
|---|---|---|---|---|
| | | 1 mD | 5 mD | 10 mD |
| Vertical | 1 mD | 4.25 | 3.12 | 3.05 |
| | 5 mD | 4.29 | 2.68 | 2.51 |
| | 10 mD | 2.80 | 2.84 | 2.65 |

**III. Weighted RMS misfits of deformation (cm)**

| Permeability | | Horizontal | | |
|---|---|---|---|---|
| | | 1 mD | 5 mD | 10 mD |
| Vertical | 1 mD | 3.55 | 2.79 | 2.87 |
| | 5 mD | 3.23 | 2.45 | 2.94 |
| | 10 mD | 2.64 | 2.53 | 3.02 |

**IV. RMS misfits to well temperature (°C)**

| Permeability | | Horizontal | | |
|---|---|---|---|---|
| | | 1 mD | 5 mD | 10 mD |
| Vertical | 1 mD | 20.59 | 20.63 | 20.56 |
| | 5 mD | 20.57 | 20.30 | 20.71 |
| | 10 mD | 20.83 | 20.59 | 20.70 |

**Supplementary Table 13. Misfits of model predictions with different thicknesses and properties (porosity, thermal conductivity and specific heat) of the subsidiary fault to surface displacement and temperature observations.**

| RMS misfit | Thickness (m) | | | Porosity (%) | | |
|---|---|---|---|---|---|---|
| | 50 | 100 | 150 | 18 | 20 | 22 |
| Leveling (cm) | 3.16 | 2.39 | 3.57 | 2.80 | 2.39 | 2.87 |
| InSAR (cm) | 2.84 | 2.68 | 2.65 | 2.78 | 2.68 | 3.04 |
| Weighted Misfit (cm) | 3.10 | 2.45 | 3.39 | 2.80 | 2.45 | 2.90 |
| Temperature (°C) | 21.72 | 20.30 | 20.75 | 21.63 | 20.30 | 20.74 |
| RMS misfit | Thermal Conductivity (W/(m·°C)) | | | Specific heat (J/(kg·°C)) | | |
| | 0.5 | 1 | 2 | 900 | 1000 | 1100 |
| Leveling (cm) | 2.39 | 2.39 | 2.39 | 2.39 | 2.39 | 2.50 |
| InSAR (cm) | 2.68 | 2.68 | 2.73 | 2.73 | 2.68 | 2.57 |
| Weighted Misfit (cm) | 2.45 | 2.45 | 2.46 | 2.46 | 2.45 | 2.51 |
| Temperature (°C) | 20.30 | 20.30 | 20.30 | 20.29 | 20.30 | 20.27 |

**Supplementary Table 14. Misfits of model predictions with different permeabilities of the subsidiary fault to surface displacement and temperature observations.**

**I. RMS misfits to Leveling observations (cm)**

| Permeability | | Horizontal | | |
|---|---|---|---|---|
| | | 10 mD | 20 mD | 30 mD |
| Vertical | 10 mD | 2.85 | 3.34 | 3.22 |
| | 20 mD | 2.29 | 2.39 | 2.97 |
| | 30 mD | 2.73 | 2.96 | 3.16 |

**II. RMS misfits to InSAR observations (cm)**

| Permeability | | Horizontal | | |
|---|---|---|---|---|
| | | 10 mD | 20 mD | 30 mD |
| Vertical | 10 mD | 2.64 | 3.01 | 2.62 |
| | 20 mD | 3.08 | 2.68 | 2.99 |
| | 30 mD | 2.77 | 2.79 | 2.61 |

**III. Weighted RMS misfits of deformation (cm)**

| Permeability | | Horizontal | | |
|---|---|---|---|---|
| | | 10 mD | 20 mD | 30 mD |
| Vertical | 10 mD | 2.81 | 3.27 | 3.1 |
| | 20 mD | 2.45 | 2.45 | 2.97 |
| | 30 mD | 2.74 | 2.93 | 3.05 |

**IV. RMS misfits to well temperature (°C)**

| Permeability | | Horizontal | | |
|---|---|---|---|---|
| | | 10 mD | 20 mD | 30 mD |
| Vertical | 10 mD | 21.20 | 20.60 | 20.67 |
| | 20 mD | 20.76 | 20.30 | 20.57 |
| | 30 mD | 20.78 | 21.03 | 21.09 |

**Supplementary Table 15. Misfits of model predictions with different scenarios of upwelling heat flux to surface displacement and temperature observations.**

| Scenario I: RMS misfit | Heat fluid rate (kg/s) | | | | | |
|---|---|---|---|---|---|---|
| | 1 | 5 | 10 | 20 | 50 | 100 |
| Leveling (cm) | 2.39 | 2.39 | 2.39 | 2.50 | 2.78 | 2.70 |
| InSAR (cm) | 2.73 | 2.73 | 2.68 | 2.73 | 2.79 | 3.12 |
| Weighted Misfit (cm) | 2.46 | 2.46 | 2.45 | 2.55 | 2.78 | 2.78 |
| Temperature (°C) | 20.30 | 20.30 | 20.30 | 20.30 | 20.34 | 20.63 |
| **Scenario II: RMS misfit** | **Heat fluid rate (kg/s)** | | | | | |
| | 1 | 5 | 10 | 20 | 50 | 100 |
| Leveling (cm) | 2.48 | 2.48 | 2.49 | 2.49 | 2.50 | 2.58 |
| InSAR (cm) | 2.76 | 2.76 | 2.75 | 2.75 | 2.73 | 2.64 |
| Weighted Misfit (cm) | 2.54 | 2.54 | 2.54 | 2.54 | 2.55 | 2.59 |
| Temperature (°C) | 20.27 | 20.27 | 20.27 | 20.27 | 20.30 | 20.60 |

Scenario I: only the normal-slip fault serving as the heat conduit; Scenario II: both of the normal-slip fault and strike-slip subsidiary fault serving as the conduit.

**Supplementary Table 16. Misfits of model predictions with different properties of water to surface displacement and temperature observations.**

| RMS misfit | Density (kg/m³) | | | Viscosity (Pa·s ) | | | | Compressibility (Pa⁻¹ ) | | |
|---|---|---|---|---|---|---|---|---|---|---|
| | 800 | 900 | 1000 | $1.5\times10^{-4}$ | $2\times10^{-4}$ | $2.5\times10^{-4}$ | $3\times10^{-4}$ | $3.7\times10^{-10}$ | $4.2\times10^{-10}$ | $4.7\times10^{-10}$ |
| Leveling (cm) | 2.42 | 2.65 | 2.39 | 3.39 | 2.57 | 2.39 | 3.03 | 3.67 | 2.39 | 3.18 |
| InSAR (cm) | 3.39 | 2.60 | 2.68 | 2.58 | 2.84 | 2.68 | 2.59 | 4.20 | 2.68 | 2.79 |
| Weighted Misfit (cm) | 2.61 | 2.64 | 2.45 | 3.23 | 2.62 | 2.45 | 2.94 | 3.78 | 2.45 | 3.10 |
| Temperature (°C) | 20.36 | 20.30 | 20.30 | 20.69 | 20.42 | 20.30 | 21.21 | 20.42 | 20.30 | 20.96 |
| **RMS misfit** | **Thermal Conductivity (W/(m·°C) )** | | | **Specific Heat (J/(kg·°C))** | | | | | | |
| | 0.58 | 0.68 | 0.78 | 3900 | 4200 | 4500 | 4800 | | | |
| Leveling (cm) | 2.39 | 2.39 | 2.39 | 2.66 | 2.39 | 2.62 | 2.80 | | | |
| InSAR (cm) | 2.68 | 2.68 | 2.68 | 2.51 | 2.68 | 2.41 | 2.99 | | | |
| Weighted Misfit (cm) | 2.45 | 2.45 | 2.45 | 2.63 | 2.45 | 2.58 | 2.84 | | | |
| Temperature (°C) | 20.30 | 20.30 | 20.30 | 20.55 | 20.30 | 20.53 | 20.44 | | | |

**Supplementary Table 17. Variations of RMS misfits of displacement and temperature in model calibration.**

| Formation | Parameter | Variation of RMS misfit[a] | |
|---|---|---|---|
| | | Displacement | Temperature |
| Reservoirs | Porosity | 33% | 10% |
| | Permeability of the upper reservoir | 31% | 6% |
| | Permeability of the lower reservoir | 36% | 7% |
| | Biot coefficient | 2% | 0 |
| | Thermal expansion coefficient | 16% | 0 |
| | Thermal conductivity | 8% | 1% |
| | Specific heat | 17% | 3% |
| Feeder fault | Thickness | 22% | 4% |
| | Porosity | 16% | 2% |
| | Permeability | 24% | 12% |
| | Young's modulus | 0 | 0 |
| | Poisson's ratio | 0 | 0 |
| | Thermal expansion coefficient | 0 | 0 |
| | Thermal conductivity | 5% | 1% |
| | Specific heat | 2% | 0 |
| Boundary fault | Thickness | 23% | 7% |
| | Porosity | 9% | 2% |
| | Permeability | 31% | 3% |
| | Thermal conductivity | 0 | 0 |
| | Specific heat | 0 | 0 |
| Subsidiary fault | Thickness | 28% | 7% |
| | Porosity | 16% | 6% |
| | Permeability | 25% | 4% |
| | Thermal conductivity | 0 | 0 |
| | Specific heat | 2% | 0 |

[a]The variation are calculated with the RMS misfits of simulated surface displacement and reservoir temperature to observations (Supplementary tables 4-14) based on the expression, $(R_{max} - R_{min})/R_{max}$, where $R_{max}$ and $R_{min}$ are the maximum and minimum RMS misfits, respectively.
